# Supplementary material for: Common and distinct neural correlates of social interaction processing and theory of mind in narratives
Source: Nat Commun. 2026 Apr 4;17:4830. doi: 10.1038/s41467-026-71151-2 (PMC13223265; doi:10.1038/s41467-026-71151-2)
Supplement: Supplementary file 1 — Supplementary information [file 41467_2026_71151_MOESM1_ESM.pdf]

Supplementary Methods

Annotations of social interactions

To test the robustness of the neural correlates of social interaction processing, four researchers, including the first author, independently annotated the narratives in terms of social interactions. We annotated one sentence part as having social interactions if and only if it involves more than one character and they have communications or physical interactions. We generated consensus labels in the same way as for ToM demands. In GLM analysis, we again created two binary regressors from the annotations, one for social interactions, the other for no social interactions. A contrast between the estimates of the two regression coefficients revealed the effects of social interactions.

Support vector machine (SVM) classification of social interactions

Modeling the social interaction annotations by two binary regressors allowed us to compare the whole-brain neural correlates of social interactions and no social interactions and test the generalizability of the pattern difference across modalities. Specifically, we performed an SVM analysis where the inputs or features were each participant's whole-brain regression coefficient maps (beta maps) and the outcomes or targets were labels of "social interactions" and "no social interactions". Two linear SVM classifiers were trained for the two modalities separately, with box constraint parameter $C = 1$. Performance of the classifier within each modality was evaluated through five-fold leave-whole-participant-out cross validation (i.e., if the social interaction map of one participant was in the left-out fold, their no social interaction map was also in the left-out fold). Performance across modalities was evaluated by applying the model trained on the entire sample in one modality to the other modality. All classifications took the form of two-alternative forced choices (2AFC), where each time a classifier was presented with two beta maps from one participant and judged which one was the social interaction map. We evaluated classification performance by two metrics: 1) classification accuracy, which is the number of hits/true positives and correct rejections/true negatives over the number of all classifications; 2) *Cohen's d*, which is calculated as the ratio of the mean of the differences of distance from the hyperplane between two beta maps over their standard deviations, and which can serve as an unbiased estimate for effect sizes.

Trial progress

As can be seen from Figure S1, for both the fMRI experiment and the online experiment, each experimental trial starts with a fixation period whose duration was selected from a uniform

distribution between 2 and 8 seconds and rounded to the nearest integer. The fixation durations were predetermined before the experiment for each trial to ensure the total duration of the fMRI experiment stayed constant. The narrative presentation period was the key time window where all regressors about social interactions and ToM were built. The online experiment had a longer narrative presentation period because we added 1.5 seconds of blank stimuli to the end of each trial, in order for participants to be able to finish their moment-by-moment ratings. The rating period was used to collect ratings about current feelings and expectations about the contents of the narratives in the fMRI experiment, and the average ratings of social interactions or ToM in the online experiment. Ratings in the rating period were not analyzed or reported in the current article.

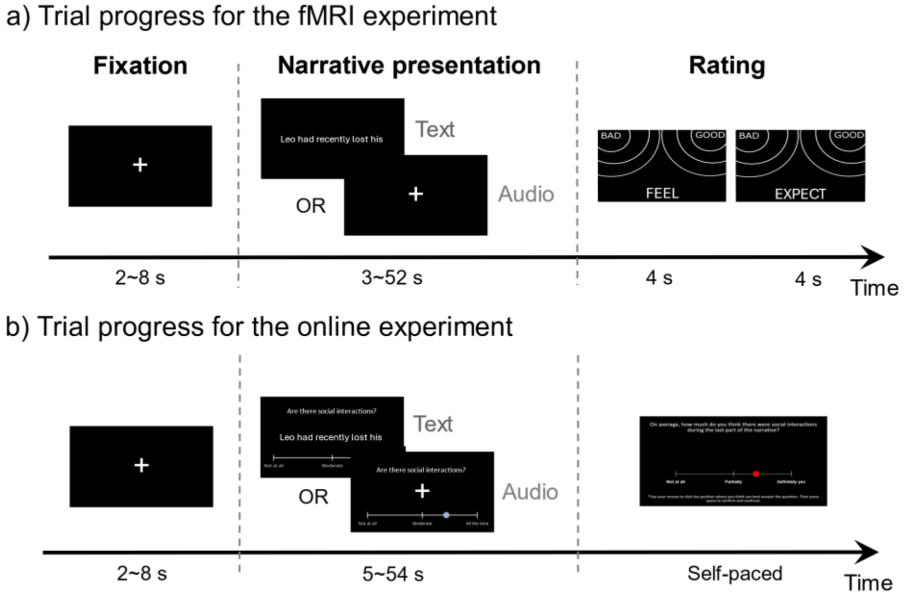

Figure S1. Trial progresses for the fMRI experiment (a) and the online behavioral experiment (b). Gray dashed lines indicated boundaries between periods. The period names, Fixation, Narrative presentation, and Rating, applied to both panels.

Behavioral ratings of action-related features

Method Background

Actions in the narratives are a class of features that may confound the observed effects of social interactions and ToM, including intentional actions, all actions, and biological motion. Intentional actions are actions that involve clear motivations and a certain level of planning, and that are congruent with the context. Theoretically, social interactions are mostly intentional (e.g., walking to a friend and asking for a favor), and intentional actions can serve as needs or “affordances” for ToM. The neural correlates of observing intentional actions have typically been

studied in contrast to observing accidental actions, actions that violate the normal intentions inferred from the context (e.g., walking around a corner alone and hitting the wall). Most studies along this line used audiovisual stimuli[1-3], where intentions of actions can be easily conveyed by lower-level features such as visual scene contexts, eye gaze directions, motion directions. They found that intentions of actions are processed in the inferior frontal gyrus, medial prefrontal cortex, posterior superior temporal sulcus, and so on. One study used short sentences to describe intentional and accidental actions, where each sentence contained only one action and whether it was intentional or accidental was determined by participants' judgments[4]. In our narratives, not every sentence contains actions, so the sum of intentional and accidental actions can form another feature, which we call "all actions" (as compared to no actions).

Biological motion are actions that have explicit descriptions of the motions of human bodies or body parts (e.g., "run", "attack", "fall"). They have been shown to activate posterior superior temporal sulcus, precuneus, premotor cortex, and inferior frontal gyrus, not only as audiovisual presentations but also as verbal descriptions[5]. Theoretically, biological motion are not a necessary condition for social interactions (e.g., eye contacts can happen without explicit motions), but some types of social interactions can involve biological motion (e.g., physical interactions). It is possible that biological motion are correlated with social interactions and are confounding the association between social interactions and neural activity.

The judgments about whether a verbally described action is intentional, compared to audiovisual presentations, can be very ambiguous (e.g., "the man dropped an object that appeared to be a knife"; also see[6]), as well as biological motion (e.g., actions of "agreeing" and "explaining"). Therefore, we quantified intentional actions and biological motion in our narratives by asking for the judgments of independent groups of participants from online studies.

Participants

A total of 209 participants took part in our online experiments from Prolific.com. The institutional review board (IRB) of Dartmouth College approved to conduct the study (CPHS STUDY00032930), and all participants provided informed consent. Each participant was compensated $10 USD after they completed the study. Of these, two participants failed at least two of the eight attention check questions, and their data were excluded from further analysis. In the remaining sample, 101 participants made judgments about intentional actions and accidental actions (52 females, 48 males, 1 with undisclosed sex; mean age = 34.38 yrs (SD = 6.62)). 106 participants judged about biological motion (51 females, 53 males, 2 with undisclosed sex; mean age = 33.27 yrs (SD = 6.64)). The sex of participants was determined based on self-report and was not considered in the study design. All participants have English as their native language and

have no literacy difficulties. All participants have an approval rate (proportion of valid data from studies they have taken part in) of over 98% on Prolific.

Stimuli and experimental procedures

Because judgements about intentions require deliberations about contextual information and the true intention of characters, it is not feasible to ask for continuous ratings about intentions in the way we did for social interactions and ToM. Instead, we broke down the narratives into smaller parts and asked participants to provide one judgment for each part. Many sentence parts in our narratives are long and involve several clauses, each of which has its own subject and action verb(s) and can be different in their action features. Thus, the parts used for action ratings are shorter and defined as "action parts", each of which should have only one subject and one or one sequence of predicates; an object is not necessary; the subject should be either explicit or able to be clearly inferred from the sentence structure; "one sequence of predicates" should be a clear continuous sequence or simultaneous combinations of actions indicated by a parallel structure (e.g., "fell to his knees and sobbed") or infinitive clause (e.g., "followed him here to confess her love"). For a full list of action parts, please see "Divisions of sentence parts and action parts".

Potential participants found our experiments on Prolific.com and used the link to take part in the experiment. After giving consent, they received instructions on how we defined intentional actions and accidental actions, or biological motion, as well as a few examples of each type. Then for each third of a narrative, they read it from the start to the end and provided their judgments for each action part in the original orders. The orders of narratives were randomized for each participant. The intentional action group judged each action part as having "intentional action", "accidental action", or "no action", while the biological motion group judged "biological motion" or "no biological motion". In the middle of each narrative, there was an attentional check question where participants needed to choose an option following the instruction. Each participant was reimbursed with $10 after they completed the whole experiment.

Behavioral ratings of multi-person presence

Method Background

In controlled experiments about perceiving social interactions presented by audiovisual stimuli, researchers usually controlled for the number of persons/agents present in a scene[7,8], in order to rule out the contributions of lower-level features to neural activity, such as perceiving bodies and faces[9]. It was less often done in naturalistic studies of processing social interactions[10-12]. In naturalistic narrative stimuli, it is hard to determine the boundary of a "scene" and in many

scenes, it is unclear how many people are "present" (by contrast, it is clear from a visual presentation as long as there are not too many people). Thus, instead of annotating the narratives on the number of people present, we aim for annotating the presence of multiple (more than one) people. To overcome the limit that there may not be clearcut "scenes", we collected continuous ratings for "multi-person presence" from an online study.

Participants

A total of 106 participants took part in our online experiment from Prolific.com. The IRB of Dartmouth College approved to conduct the study (CPHS STUDY00032930), and all participants provided informed consent. Each participant was compensated $8 USD after they completed the study. We used the same criteria for participant filtering as the experiment for social interaction and ToM ratings. Three participants provided the same ratings throughout the experiment, so their data were excluded from analysis, but all participants correctly answered at least half of the attention check questions and all participants had their data saved. The remaining 103 participants with valid data (50 females, 53 males) are all healthy adults (19-45 years old, M = 32.02, SD = 6.53) whose native language is English and who have no literacy difficulties. The sex of participants was determined based on self-report and was not considered in the study design. All participants have an approval rate (proportion of valid data from studies they have taken part in) of over 98% on Prolific.

Stimuli and experimental procedures

The stimuli were presented in the exact same way and orders as the behavioral ratings of social interactions and theory of mind (see "Behavioral ratings of social interactions and theory of mind" in main text for details). Participants' tasks also shared the same structure; the only difference is the ratings requested: Here, all participants answered the question: "*Are there multiple people? (How much do you think there are multiple people in a scene at this moment?)*". The question in the bracket only appeared in the practice trials. The three anchors of the visual analog scale are "*Not at all*", "*Partially*", and "*Definitely yes*". Each participant was reimbursed with $8 after they completed the whole experiment.

Data collection and quantification

We used the same parameters as the experiments of social interaction and ToM ratings in data collection (such as the sampling rate and data values). We also followed the same data cleaning procedures, including removing low-quality data, setting missing values, and resampling the ratings to one sample per 230ms. In calculating a group-level median of multi-person presence ratings, we excluded participants' data that had a correlation with the medians of all other participants' ratings lower than 0.2 (n = 18).

Supplementary Results

Neural correlates of social interaction processing as researchers' annotations

The four researchers largely agreed with each other on their annotations about social interactions (pairwise Pearson's correlations [.66, .78] (*M* = .71; Fleiss' kappa = .71). In fMRI analysis, we again found very similar effect maps across modalities (*r* = .69; Figure S2a). Across two modalities, the common regions being activated were largely the same as the analysis using online participants' ratings, including TPJ, STS, dmPFC, and so on. Indeed, the unthresholded conjunction maps estimated from the two methods were highly similar (*r* = .86). Those results provided evidence that the neural correlates we found were invariant across measurements, no matter whether they were from researcher annotations or from online participants' ratings.

In the SVM analysis, we found large pattern differences between the "social interaction" and "no social interaction" beta maps, as suggested by the high within-modality accuracies (> 97%) and effect sizes (> 1.8). More importantly, the classifiers trained in one modality also worked well in the other (accuracies > 90%, *Cohen's d* > 1.3, Figure S2b). It indicated that not only general activation patterns but also the differences between patterns caused by social interactions were similar across modalities. Together, those results provided strong evidence that the same neural circuits responded to social interaction information in the narratives both when they were visually and auditorily presented.

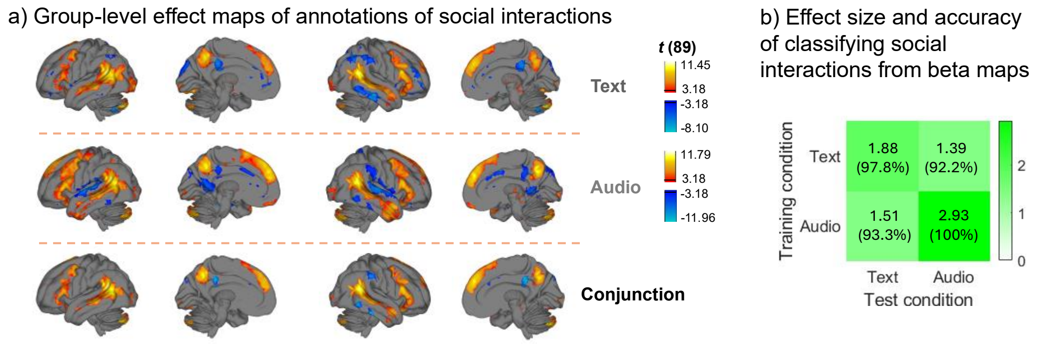

Figure S2. Neural correlates of social interactions as researchers' annotations. a) Contrast map of social interaction versus no social interaction. b) The confusion matrix of a support vector machine (SVM) analysis to predict the labels of whole-brain beta maps (social interactions or no social interactions). Models trained in one modality (Text or Audio) were tested in both modalities. Performance was estimated by classification accuracy (in brackets) and Cohen's *d.* This figure shows that annotations of social interactions had similar neural correlates with those of continuous

ratings of social interactions, and these neural correlates were generalizable between the Text and Audio modality.

Neural correlates of ToM engagement and ToM demands

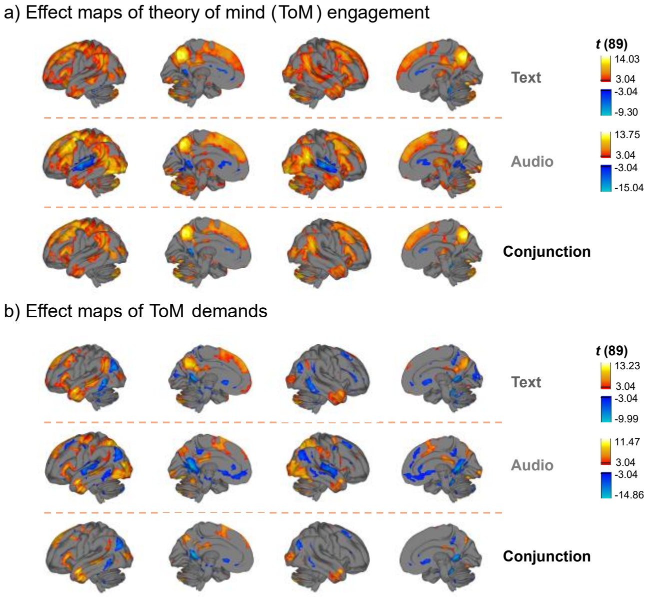

Figure S3. Neural correlates of ToM engagment (a) and ToM demands (b) in two modalities separately and their conjunctions. Each set of maps was thresholded at FDR $q < .01$. Across modalities, the neural correlates of ToM were not as similar as social interaction processing but still in the moderate range (spatial $r = .58$ for ToM engagement, spatial $r = 52$ for ToM demands). In both modalities, ToM demands consistently activated larger canonical ToM regions.

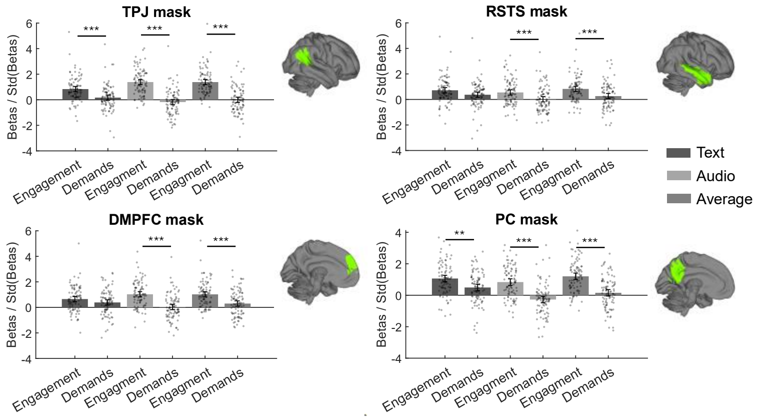

Figure S4. Comparison of effect sizes of self-reported ToM engagement and ToM demands. This figure shows comparisons in the brain masks defined by the ToM group map[13] (see Methods for details) and uses the same statistical tests as in Figure 4b (N = 90 participants for each

comparison). All raw p values from left to right, up to bottom: ***: $p < .001$, **: $p < .01$ (with Bonferroni correction). For all significant comparisons that have a correct p value lower than .001, an exact p value is not possible to estimate because of the precision limit of the Bootstrap procedure. For the other three comparisons, raw p values are (from left to right, up to bottom) .012, .043, .0002. Abbreviations: RSTS – right STS. In most comparisons, ToM engagement had significantly larger effect sizes (all Mean differences > .57); in RSTS and DMFPC in the Text modality, the differences were not significant (both Mean differences < .35).

Neural correlates of social interaction processing and ToM

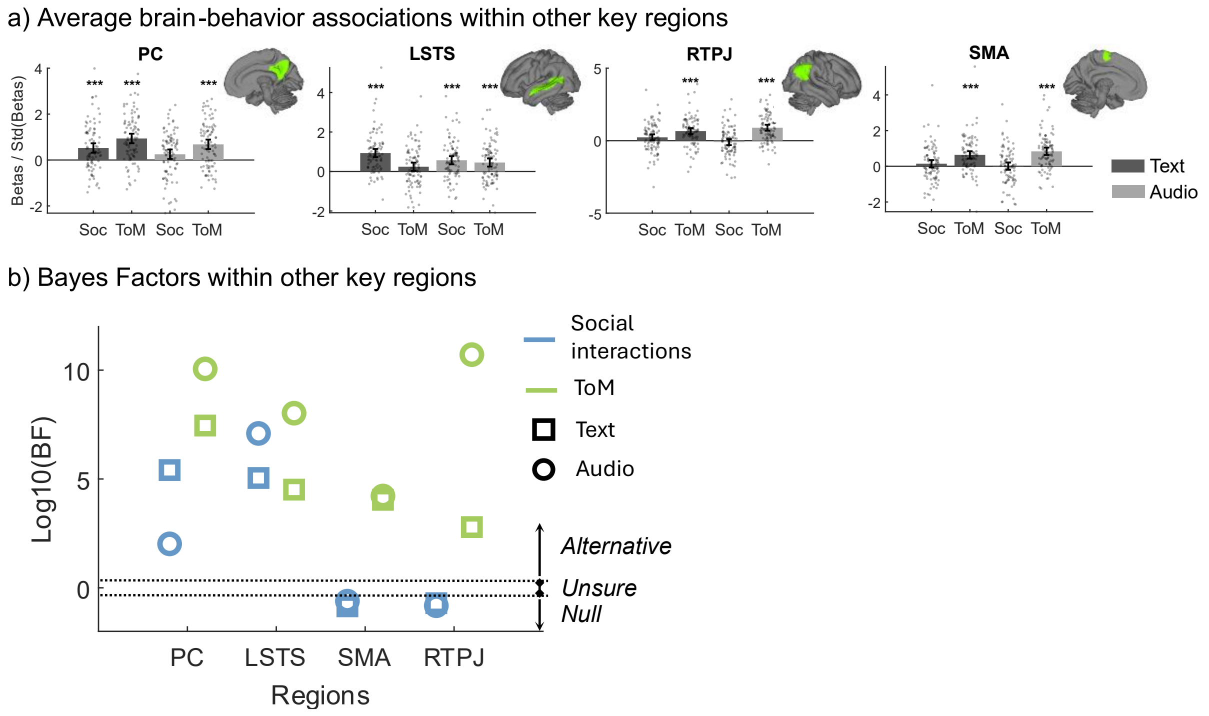

Figure S5. Average betas and Bayes Factors in selected brain regions. a) complements Figure 5c and b) complements Figure 6b. All statistical tests are the same and all conventions follow those two figures (N = 90 participants for each bar). All raw p values from left to right in a): 3.34e-6, 0, .019, 4.95e-9, 0, .025, 5.36e-7, 4.24e-5, .03, 1.21e-8, .35, 5.40e-13, .18, 5.96e-8, .93, 5.50e-12 (0 indicates a p value lower than 1e-13). ***: $p < .001$ with Bonferroni correction. In the precuneus (PC) and left superior temporal sulcus (LSTS) mask, both social interactions and ToM had significant effects (except social interactions under the Audio modality (uncorrected p = .019) and ToM under the Text modality (uncorrected p = .025) whose effects were not significant under Bonferroni Correction). In the right temporo-parietal junction (RTPJ) and supplementary motor area (SMA) mask, only ToM had significant effects.

Other social cognitive features

In this section, we evaluated several social cognitive features other than social interactions and ToM in the narratives, compared them to social interactions and ToM on both behavioral and neural level. We assessed whether any of those features confounded the relationships between social interactions/Tom and the neural activity, and whether any feature explained neural activity better than social interactions/ToM in pre-defined brain masks.

Intentional actions and all actions

We quantified the levels of intentional/accidental actions as the proportions of participants who gave an "intentional action"/"accidental action" judgment to each action part (see Supplementary Methods for details). Across narratives, levels of intentional actions were not correlated strongly with either social interactions (for the Text modality, r = .11; for the Audio modality, r = -.02) or ToM (rs = .03, .03, for the two modalities), neither were accidental actions (all absolute rs <.11). Because many previous studies focused on the contrast between intentional and accidental actions, we also correlated the difference between intentional and accidental actions with social interaction and ToM ratings, which did not yield strong correlations, either (all absolute rs < .08). Besides, the sum of intentional and accidental actions, or all actions, were only weakly correlated with social interactions or ToM ratings (all absolute rs < .14). After checking the ratings with narrative texts, we found that there are parts of narratives where both social interaction ratings and intentional action ratings are high (e.g., "He grabbed her by the arms and started shaking her") or low (e.g., "She knew how much it would hurt Amy to know the truth"). However, there are also parts where social interaction ratings are high but intentional action ratings are low (e.g., (within the scene of Zach interacting with another one) "Zach realized his mistake in judgment and shifted his anger") or vice versa ("James made his way to Matthew's house to meet back with his son"). It implies that intentional and accidental actions may be lower-level features that fluctuate relatively independent of social interaction scenes and cannot fully explain the effects of social interactions or ToM.

Because of the low correlations on the behavioral level, we investigated the independent neural correlates of processing intentional actions or all actions in narratives by fitting separate models for each. We mean-centered ratings across the four narratives in each modality and used them as continuous regressors in voxelwise GLM analysis. On the group level, we found that the neural correlates of processing intentional actions were similar across modalities (r = .66 between two unthresholded t maps). Across modality, the common positive neural correlates were found

in bilateral medial temporal lobes (MTL), posterior cingulate cortex (PCC), anterior cingulate cortex, bilateral inferior temporal gyrus, and other small clusters. The common negative neural correlates were in bilateral STS, left TPJ, bilateral PC, left IFG, left premotor cortex, and bilateral dmPFC (Figure S6a). The neural correlates of processing all actions were moderately similar across modalities (r = .45) but very similar to those of processing intentional actions (rs = .94 and .76 for Audio and Text modality, respectively). Across modalities, processing all actions is positively associated with neural activity in bilateral MTL, PCC, right TPJ, left inferior temporal gyrus, and right IFG; it is negatively correlated with neural activity in left STS, left TPJ, bilateral PC, left dmPFC, left IFG, and left premotor cortex (Figure S6b). Some of the negative associations found here were in the key regions associated with social interactions and ToM. This suggests that intentional actions or all actions cannot explain the neural correlates of social interactions and ToM. Together with the behavioral correlations, it supports the view that they are not confounding the effects of social interactions or ToM.

Biological motion

Similar to intentional actions and all actions, we quantified the levels of biological motion as the proportions of participants who gave a “biological motion” judgment to each action part. We again found that levels of biological motion in narratives were only weakly correlated with social interactions or ToM ratings (all absolute rs < .11, in both modalities). On the neural level, processing biological motion elicited moderately similar activity patterns across modalities (r = .59). Across modalities, the common positive regions included right TPJ, left MT+, bilateral superior parietal lobule, PCC, bilateral MTL, left supramarginal gyrus, and left TPJ. The common negative regions included right STS, bilateral PC, left IFG, left dmPFC, and left TPJ.

It is worth noting that although conceptually different, ratings about intentional actions, all actions, and biological motion were highly similar (all pairwise rs >= .6). Correspondingly, they also have similar neural correlates that were different from those of processing social interactions and ToM. Thus, the class of action-related features cannot confound the effects of social interactions and ToM, and whether to control for them should not affect the specificity of our results.

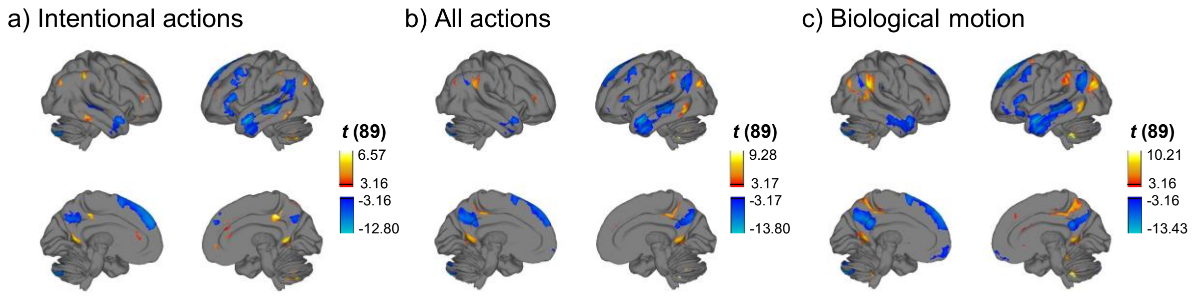

Figure S6. Neural correlates of processing Intentional actions (a), All actions (b), and Biological motion (c) as conjunctions across two modalities. Those neural correlates partially overlapped with those of social interactions and ToM, but also had an opposite sign in other regions, suggesting their effects were different from those of social interactions and ToM. The three maps were thresholded at FDR $q < .01$ independently.

Multi-person presence

Features of ratings

Participants' ratings about multi-person presence are highly reliable, as indicated by the large split-half correlations ([.94, .97], $M = .96$; estimated from 2000 permutation samples and corrected by the Spearman-Brown formula[14,15]). Also, different participants' ratings were moderately similar to each other (median of pair-wise correlations = .38). Those results support the validity of using the medians of all participants' ratings as a normative measurement of multi-person presence.

To quantify the relationship between multi-person presence and social interactions and ToM, we correlated the medians of those ratings across time under two modalities separately. As expected, multi-person presence highly correlated with social interactions (*r*s = .70, .81, for Audio and Text modality, respectively; Figure S7a). Also, it correlated weakly to moderately with ToM (*r*s = .26, .29, for Audio and Text modality, respectively). More importantly, ratings about multi-person presence were rarely lower than ratings about social interactions (on only 16.6% of all story timepoints; Figure S7a); even when social interaction ratings were higher, they were very close to multi-person presence ratings (at all those time points, the differences between two ratings were always below 14.2 out of 100, and the average difference was 3.2; they were in contrast to a maximum difference of 73.3 and average of 25.2 at timepoints where multi-person presence ratings were higher). It is consistent with the definition of social interactions which involves multi-person presence, such that multi-person presence is a necessary but not sufficient condition for social interactions.

Neural correlates of processing multi-person presence

We investigated the neural correlates of processing multi-person presence by using medians of online participants' ratings as the only regressors in GLM analysis. On the group level, the neural correlates are similar across modalities (spatial r = .64). Multi-person presence is positively associated with neural activity in bilateral TPJ, STS, PC, dmPFC, and dorsolateral prefrontal cortex; there were rarely any common negative neural correlates (Figure S7b). Noticeably, the whole-brain neural correlates are very similar to those of social interactions ((spatial rs = .86, .96 for Audio and Text modality, respectively) and ToM (spatial rs = .83, .75). It suggests that multi-person presence may be confounding the neural correlates of social interaction processing and ToM.

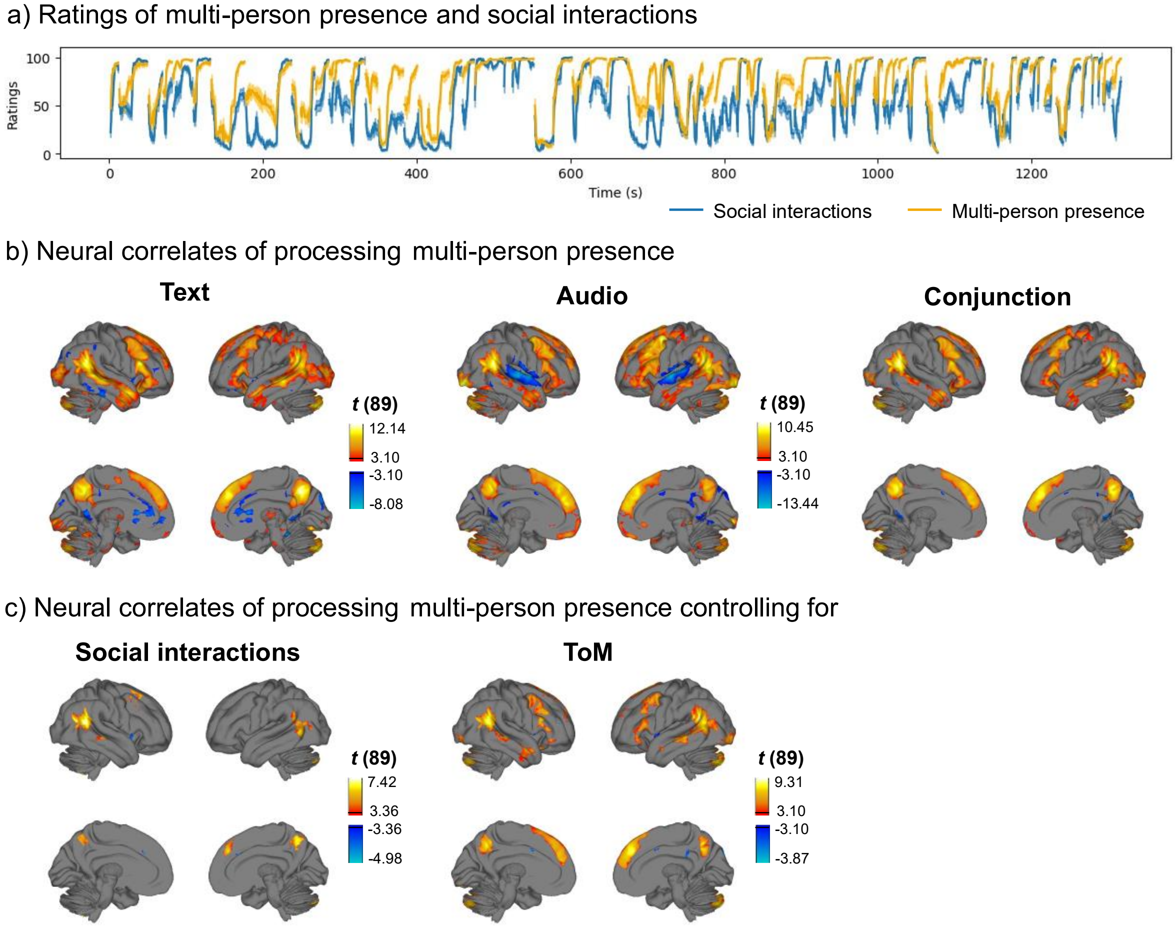

Figure S7. Results about the presence of multiple people (multi-person presence). a) The medians of online participants' ratings of multi-person presence and social interactions across all story timepoints. The shaded areas around the lines are simulated confidence intervals calculated by median absolute differences. Ratings of multi-person presence and social interactions were

highly correlated, with the former being higher or similar to the latter at almost all time points. b) The group-level *t* maps of processing multi-person presence, thresholded at FDR $q < .01$. They were very similar to the maps of social interactions and ToM. c) Group-level *t* maps of processing multi-person presence after controlling for social interactions or ToM, as a conjunction across two modalities, thresholded at FDR $q < .01$. Compared to its independent effects, controlling for social interactions rendered most significant voxels unsignificant, while controlling for ToM had smaller effects.

To test the potential confounding effect, we fit two other models where multi-person presence was included with social interactions or ToM. Because ratings of social interactions and multi-person presence were highly correlated, the model fit with both regressors was noisier and more unstable. It was corroborated by the lower correlations between *t* maps across modalities (spatial $r = .29$ for social interactions, spatial $r = .27$ for multi-person presence). The two regressors competed to explain neural activity, resulting in opposite neural patterns in the whole brain (spatial *r*s = -.60, -.19 for Audio and Text modality, respectively) and lower *t* values for social interactions in many voxels (Figure S8a right). Still, After controlling for multi-person presence, 58.6% (Audio) and 30.8% (Text) of the positive significant voxels associated with social interactions were still significant with the same sign, under the same threshold (Figure S8a). Although the positive neural correlates of social interaction processing “shrank”, they still covered the key regions, including bilateral STS, PC, dmPFC, IFG, and left TPJ, except right TPJ (Figure S8a). Besides, the overall spatial patterns remained relatively stable (spatial *r*s = .81 and .69 between the effect maps of social interaction processing with and without controlling for multi-person presence, for Audio and Text, respectively). By contrast, the positive neural correlates of multi-person presence shrank even more (21.6% (Audio) and 25.7% (Text) of positive significant voxels were retained; Figure S7c). Those results suggest that the effects of social interactions on neural activity cannot be fully explained by multi-person presence, and social interactions are a better predictor of neural activity in many key social cognitive regions.

When we included both ToM and multi-person presence ratings in the same model, we found almost identical neural correlates of ToM as when we included only ToM in the model (spatial *r*s = .99, .97 for Audio and Text modality, respectively; Figure S8b). 94.8% (Audio) and 83.3% (Text) of positive significant voxels associated with ToM were still significant with the same sign after controlling for multi-person presence (Figure S8b). By contrast, 57.5% (Audio) and 64.6% (Text) of positive significant voxels associated with multi-person presence were significant with

the same sign after controlling for ToM (Figure S7c). It indicates that multi-person presence cannot explain the neural activity associated with ToM, and thus cannot confound its effects.

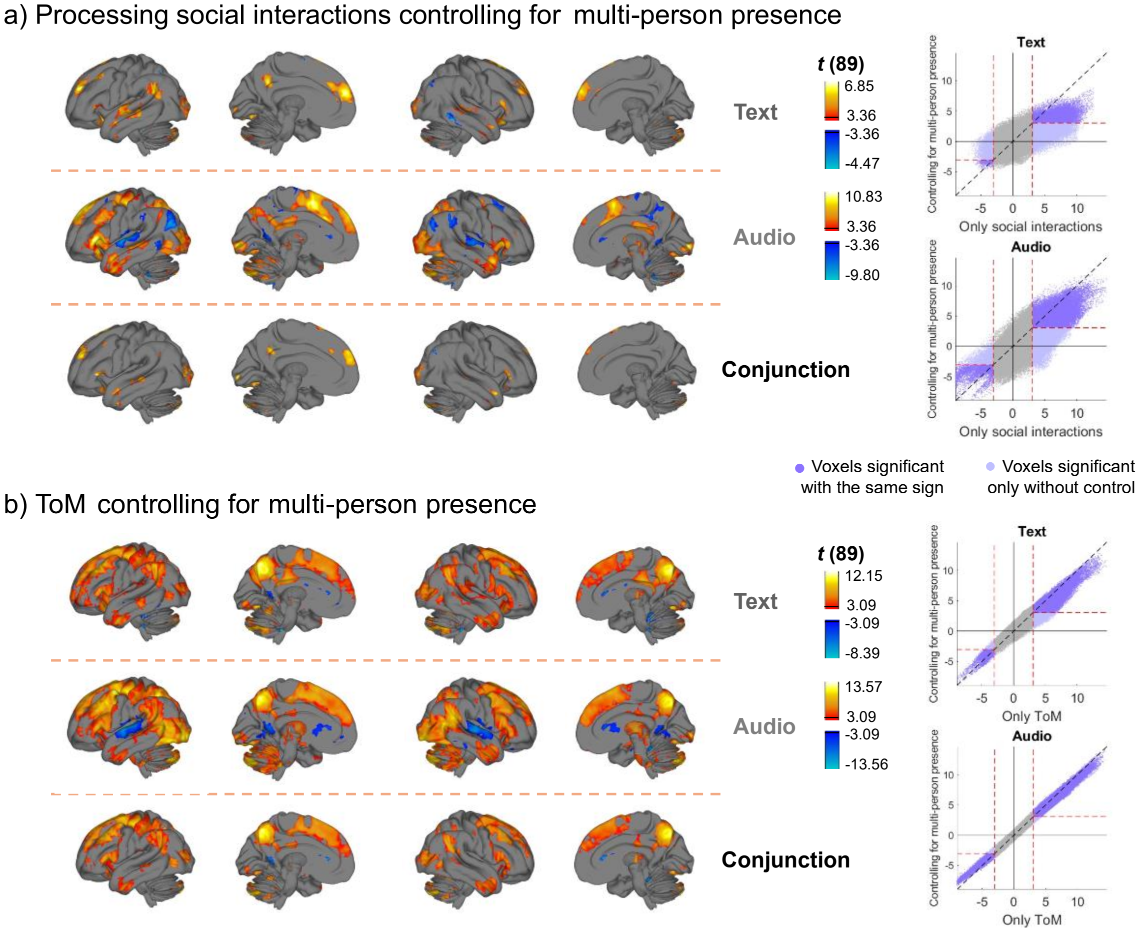

Figure S8. Neural correlates of processing social interactions (a) and ToM (b) after controlling for multi-person presence, thresholded at FDR $q < .01$. In the scatter plots on the right, t values of each voxel without and with controlling for multi-person presence were plotted along the x and y axis, respectively; red dashed lines indicate the threshold for significance; dark purple dots indicate the voxels that were significant with the same sign regardless of whether to control multi-person presence, light purple dots indicate the voxels that were significant without controls but were not significant with controls, and gray dots indicate voxels that were not significantly associated with social interactions/ToM. This figure shows that the effects of social interactions were reduced by controlling for multi-person presence but still significant in most key regions, while the effects of ToM were rarely affected.

Indoor/Outdoor scenes

In naturalistic studies of social cognition, whether the story happens indoors or outdoors is a feature that can drive neural activity in parts of the brain[16,17]. It may be related to social interactions in some stimuli[18] but not others[11]. We followed the procedure of previous studies to manually annotate whether each sentence in the narratives describes events indoors or outdoors. Because some sentences in the narratives are not about concrete events (e.g., "Linda and Amy had been best friends since kindergarten.") and thus cannot happen only indoors or outdoors, we allowed not only labels of indoors and outdoors but also labels of "ambiguous". Overall, there were more indoor scenes (48.4% of all story time points) than outdoor scenes (34.0%) in the narratives we used; the remaining small proportion (17.6%) of stories are ambiguous in terms of indoor/outdoor scenes. We took the difference between the indoor and outdoor annotations to get an estimate of the contrast feature "indoor – outdoor". This contrast feature did not correlate with ratings of social interactions (r = .03) or ToM (r = -.05) under the Audio modality but did correlate non-trivially under the Text modality (rs = .20, .27 for social interactions and ToM, respectively). It suggests that indoor/outdoor scenes may correlate with ratings of social interactions and ToM, but the correlations are not reliable across stimuli.

In GLM analysis of BOLD data, we created two regressors "indoors" and "outdoors", each of which has three values (1, 0, or 0.5; 0.5 corresponds to "unsure"). We added them to the same model, so a contrast between the two estimated slopes could reveal the neural correlates of processing stories that happen indoors versus outdoors. Those neural correlates were not similar across modalities (spatial r = .16). Under Audio modality, indoor scenes activated bilateral PC, right TPJ, and right anterior temporal lobe more strongly than outdoor scenes. Those effects were also present under Text modality, with other effects in bilateral STS, dmPFC, IFG, and left TPJ (Figure S9). Intriguingly, outdoor scenes in narratives activated right parahippocampal cortex more strongly than indoor scenes under the Text modality, consistent with previous findings using visual stimuli[19,20]. It implies some generalizability of neural responses in parahippocampal cortex across modality.

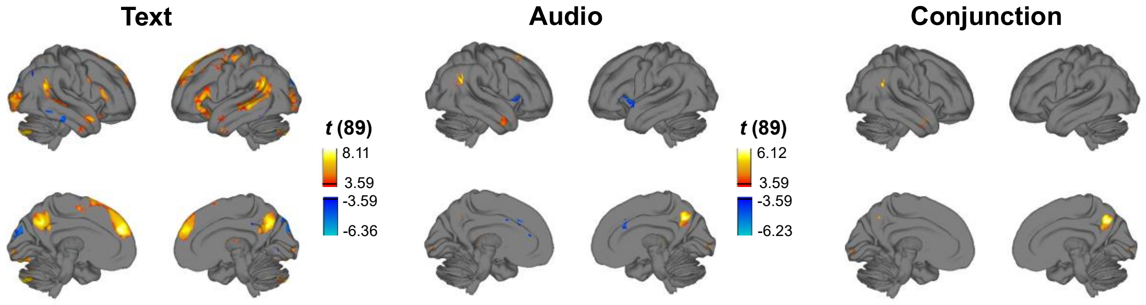

Figure S9. Neural correlates of processing indoor scenes minus outdoor scenes, thresholded at FDR $q < .01$. The positive regions overlapped with the positive neural correlates of social interaction processing and ToM. After controlling for social interactions or ToM, there were rarely any significant voxels (~0.1% of all voxels) in the conjunction map across modalities, so we did not show the effect maps here.

Because the indoor versus outdoor contrast seems to activate regions overlapping with social interactions/ToM, we also controlled for it and tested how that affected the neural correlates of social interactions and ToM. In this model, we added the difference and the sum of indoor and outdoor scenes; the difference is algebraically equivalent to the contrast in the simpler model with only indoor and outdoor scenes. We found that the *t* maps of social interactions were highly similar between controlling for indoor/outdoor scenes and not controlling (spatial *r*s = .97, .97 for Audio and Text modality, respectively), as well as for ToM (spatial *r*s = .98, .90). Almost all positive significant voxels associated with social interactions were still significant with the same sign after controlling for indoor/outdoor scenes (94.1% and 91.9% for Audio and Text modality, respectively), as well as for ToM (95.5% and 85.1%) (Figure S10). It suggests that whether to control for indoor/outdoor scenes or not rarely affects the neural correlates of social interactions and ToM, and indoor/outdoor scenes are not confounding the effects of social interactions and ToM.

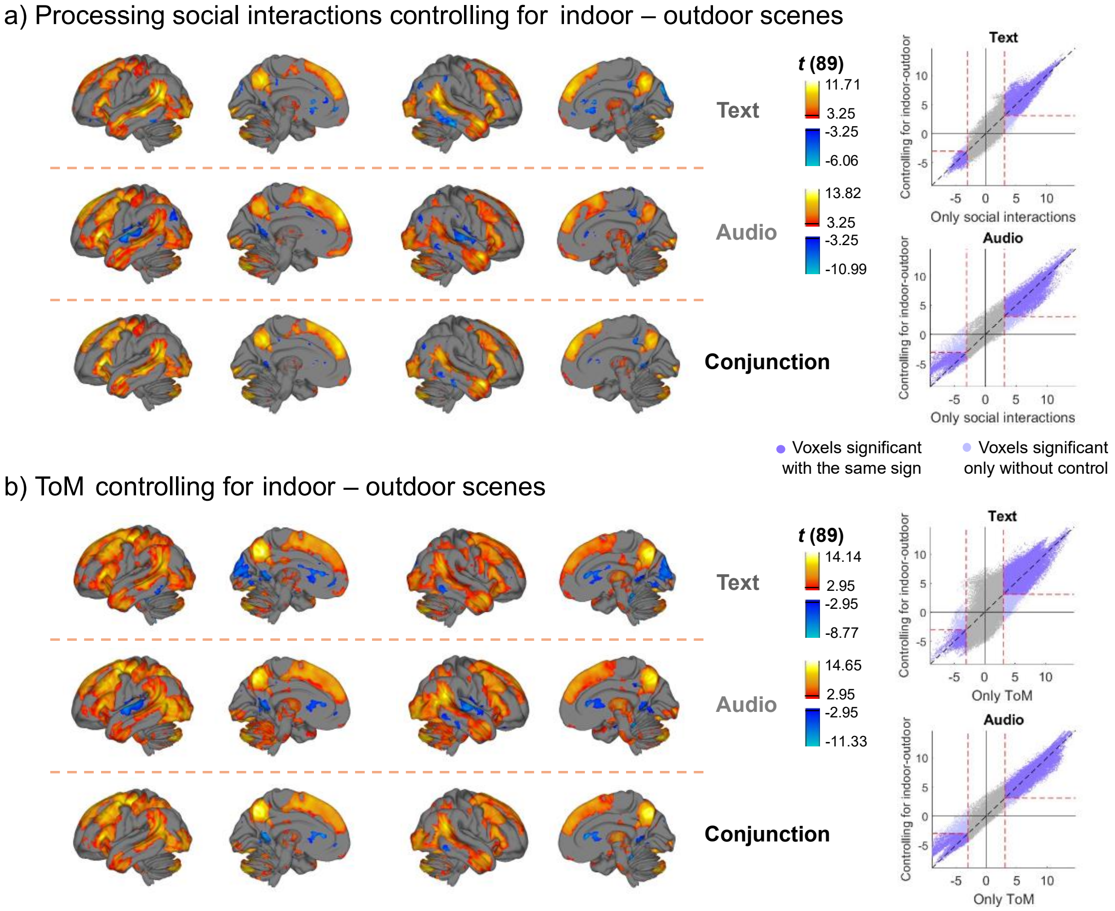

Figure S10. Neural correlates of processing social interactions (a) and ToM (b) after controlling for the contrast between indoor and outdoor scenes, thresholded at FDR $q < .01$. The brain maps on the left show that the effects of social interaction processing and ToM were rarely affected by processing indoor versus outdoor scenes. In the scatter plots on the right, t values of each voxel without and with controlling for indoor-outdoor scenes were plotted along the x and y axis, respectively; all conventions follow those of Figure S8.

Valence of narratives (Sentiment polarity)

Emotional experience during naturalistic stimuli can drive neural activity in many "social brain" regions[11,21]. To investigate whether it can confound the effects of social interactions and ToM, we annotated the valence of each sentence in the narratives to approximate an average person's emotional experience during narrative processing. Specifically, we used a BERTweet-based language model finetuned for sentiment analysis[22] to predict the sentiment polarity of each sentence in our narratives ("positive", "neutral", or "negative"). We also retained the probabilities of each label (larger probabilities for the "positive" label are approximations for more positive

valence). Across all narratives, we found most sentences to have a “neutral” label, followed by “negative” and lastly “positive”. Importantly, none of those labels or probabilities were strongly correlated with ratings of social interactions under each modality (all absolute *r*s <= .17, except “negative” label correlated with social interactions at r = -.23 under audio modality) or across all narratives (all absolute *r*s <= .04). The same is true for ToM under each modality (all absolute *r*s <= .19) or across all narratives (all absolute *r*s <= .13).

On the neural level, we estimated the neural correlates of processing positive, neutral, and negative sentences by fitting each label as a boxcar regressor in GLM analysis. We focused on the contrast between each pair of the three labels to control for the effects of processing stories. Across modalities, neither of those contrasts yielded similar neural correlates (all spatial *r*s across modalities were below 0.3), suggesting non-generalizable neural correlates of sentiment polarity (Figure S11). More importantly, under both modalities, the correlations between the contrast maps and social interactions/ToM effect maps were all below .25, except a correlation of .44 between positive versus neutral and social interactions under Text modality. Those results suggest that the neural correlates of sentiment polarity in narratives are different from those of social interactions and ToM. Together with behavioral results, it implies that valence of narratives cannot confound the effects of social interactions or ToM.

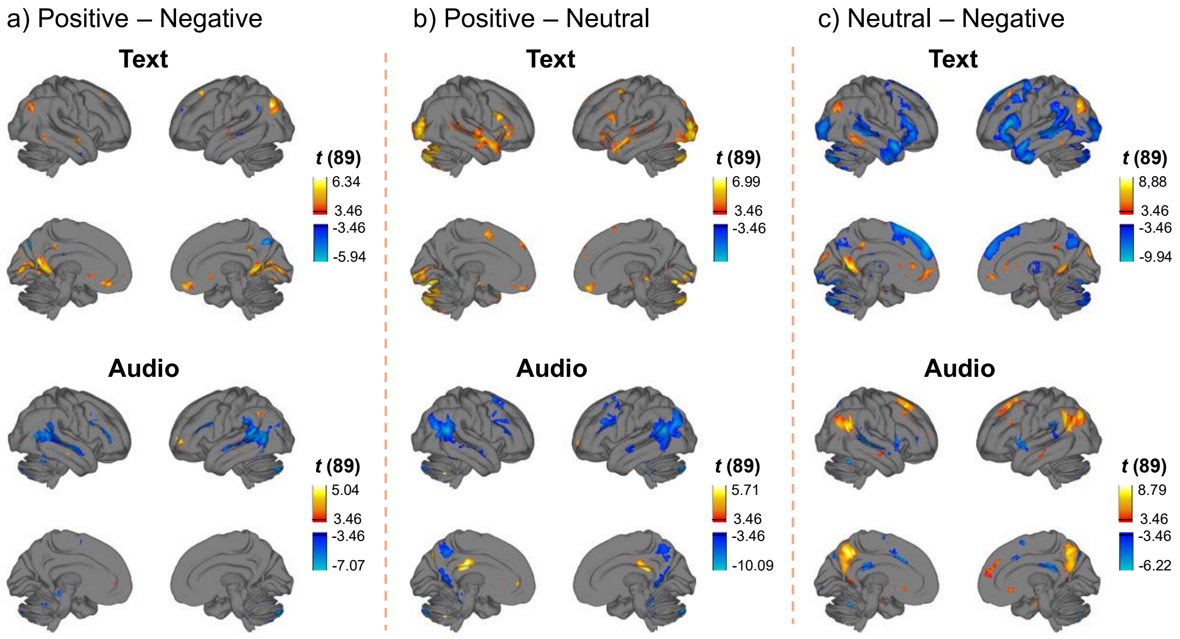

Figure S11. Neural correlates of the contrasts of processing different sentiment-label sentences. The three panels a to c show the effect maps of Positive versus Negative, Positive versus Neutral, and Neutral versus Negative, respectively, thresholded at FDR q < .01. Because maps are

different across modalities, only a few voxels showed cross-modal activations, so no conjunction maps are displayed. Across all maps, some positive regions overlapped with those of social interactions/ToM, but those patterns were not consistent across modalities or contrasts.

Comparing independent effects of various features

To investigate whether any social cognitive features other than social interactions and ToM could explain neural activity better in regions defined by previous studies, we compared their independent effects from each model. Specifically, we used the same methods as comparing ToM engagement with ToM demands (Figure 4) to compare the effect sizes of each additional feature to those of social interactions and ToM under each modality. We selected the Neurosynth-based "social interaction", "social cognition", "psts", "tom", and "mentalizing" masks as defined in the Main Text. The features that were compared included intentional actions, all actions, biological motion, multi-person presence, indoor versus outdoor scenes, positive versus negative, positive versus neutral, and neutral versus negative. Those resulted in 160 comparisons in total (Table S1), and a p value of .00031 or lower was required for a significant result under Bonferroni Corrected threshold of .05. We compared the effect sizes of social interactions and ToM with the absolute effect sizes of other features (Figure S12) to get a more conservative test of whether social interactions/ToM had stronger explanatory power regardless of signs.

We found that in many of those comparisons, social interactions and ToM had significantly larger effect sizes than other features (Table S1). Importantly, none of the individual comparisons was significantly negative (the smallest p value associated with a negative effect size difference was .012). Those results indicate that in brain regions found to be associated with social interaction processing and ToM in previous studies, social interactions and ToM ratings in the current study explain the neural activity better than or about equally well as other related social cognitive features. Given the conservativeness of the comparisons, it provides strong evidence that social interactions and ToM are the key features that drive neural activity in those regions.

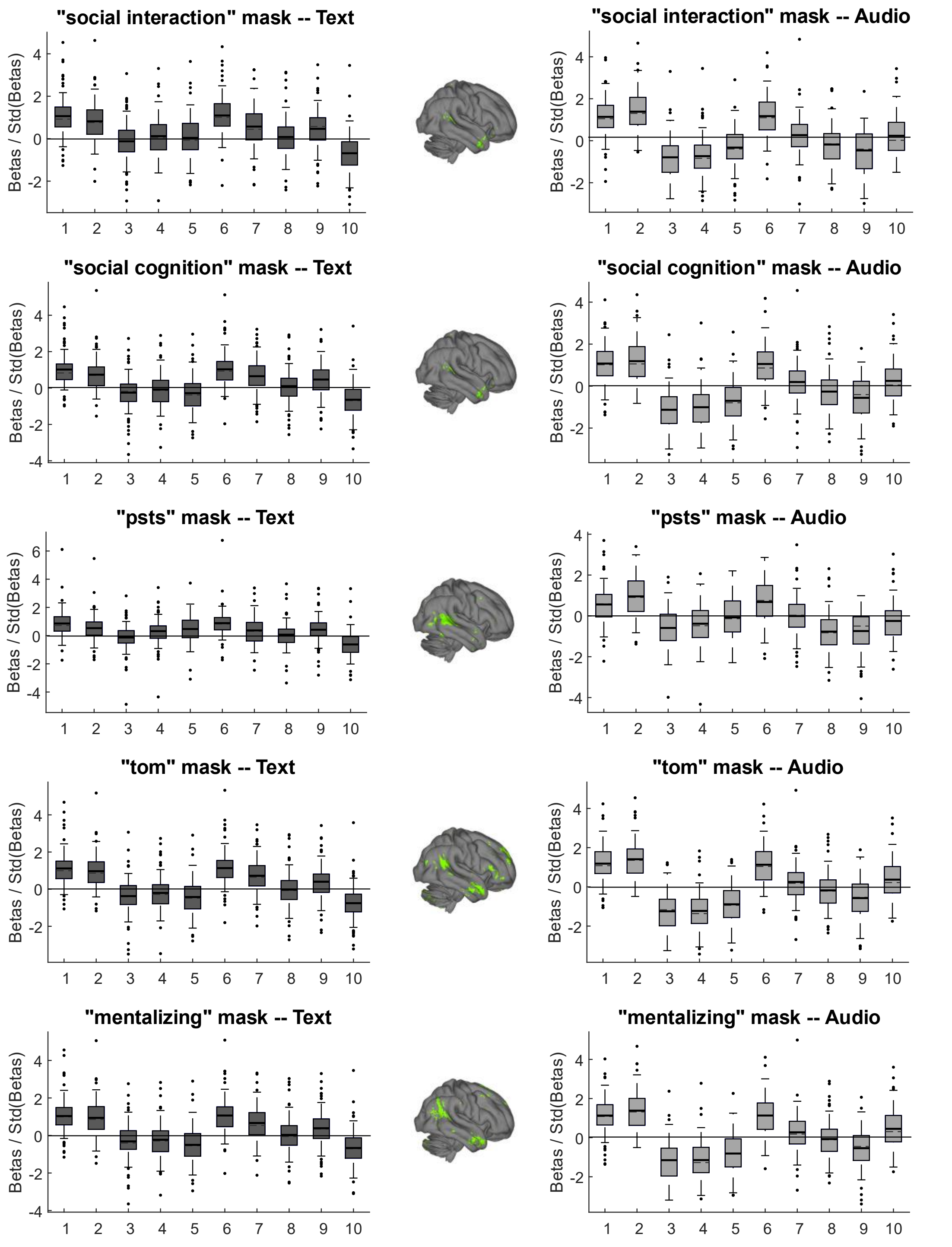

X-axis labels: 1 – Social interactions, 2 – ToM
3 – Intentional actions, 4 – All actions, 5 – Biological motion, 6 – Multi-person presence
7 – Indoor vs. outdoor, 8 – Positive vs. Negative, 9 – Positive vs. Neutral, 10 – Neutral vs. Negative

Figure S12. The effect sizes of 10 features in explaining neural activity in pre-defined brain masks associated with social interactions or ToM. The y-axes showed the individual mean regression

coefficients (betas) divided by the standard deviation across participants (*N* = 90 participants for each box). The top edges of the box are the 1st and 3rd quartile of each group of data, the whiskers extend to the most extreme data points not considered outliers (data points that are more than 1.5 interquartile range away from the 1st and 3rd quartile), and outliers are plotted individually as '*' symbols. Solid lines within boxes represent the means (Cohen's *d*), while dotted lines represent medians (in many cases they overlap in the figure). In most comparisons, social interactions and ToM had significantly larger effect sizes, while in any comparisons, none of the other feature had larger effect sizes than social interactions/ToM.

Table S1

Difference in absolute effect sizes of all pair-wise comparisons between social interactions/ToM and other social cognitive features

	Inten-tional actions	All actions	Biolo-gical motion	Multi-person presence	Indoor versus outdoor	Positive versus Negative	Positive versus Neutral	Neutral versus Negative
"social interaction" mask – Text modality								
Soc.	**0.94**	**0.95**	**1.03**	-0.03	**0.49**	**0.99**	0.60	0.38
ToM	**0.70**	**0.70**	**0.78**	-0.28	0.25	**0.74**	0.35	0.13
"social interaction" mask – Audio modality								
Soc.	0.35	0.41	**0.81**	-0.03	**0.87**	**0.96**	0.68	**0.90**
ToM	0.60	0.65	**1.06**	0.21	**1.12**	**1.20**	**0.92**	**1.15**
"social cognition mask" – Text modality								
Soc.	**0.76**	**0.93**	**0.73**	-0.01	0.37	**0.91**	0.55	0.37
ToM	0.48	**0.65**	0.45	-0.29	0.08	**0.63**	0.27	0.09
"social cognition mask" – Audio modality								
Soc.	-0.06	0.07	0.37	0.02	**0.88**	**0.81**	**0.51**	**0.83**
ToM	0.06	0.18	0.49	0.14	**1.00**	**0.93**	**0.63**	**0.94**
"psts" mask – Text modality								
Soc.	**0.73**	**0.54**	0.39	-0.02	**0.49**	**0.79**	0.44	0.24
ToM	0.40	0.21	0.06	-0.35	0.16	0.46	0.11	-0.09
"psts" mask – Audio modality								
Soc.	-0.03	0.18	**0.48**	-0.12	0.55	-0.21	-0.18	0.31
ToM	0.36	**0.57**	**0.88**	0.27	**0.95**	0.18	0.21	**0.70**
"tom" mask – Text modality								
Soc.	**0.74**	**0.91**	**0.69**	-0.02	0.39	**1.09**	**0.72**	0.36

ToM	**0.59**	**0.77**	**0.54**	-0.17	0.25	**0.94**	0.57	0.21
“tom” mask – Audio modality								
Soc.	-0.06	-0.04	0.30	0.06	**0.93**	**1.01**	**0.61**	**0.82**
ToM	0.16	0.18	0.52	0.28	**1.16**	**1.23**	**0.83**	**1.04**
“mentalizing” mask – Text modality								
Soc.	**0.72**	**0.81**	0.54	-0.03	0.37	**1.01**	**0.65**	0.37
ToM	**0.62**	**0.71**	0.44	-0.12	0.27	**0.91**	0.55	**0.27**
“mentalizing” mask – Audio modality								
Soc.	-0.02	-0.02	0.32	-0.002	**0.86**	**1.06**	**0.59**	**0.68**
ToM	0.23	0.23	0.57	0.25	**1.11**	**1.31**	**0.84**	**0.93**

Note. Bold fonts indicate values that are significant under the Bonferroni Corrected (160 comparisons) threshold of .05 (*p* values estimated from 10,000 Bootstrap samples). For most significant comparisons, an exact *p* value is not possible to estimate because of the precision limit of the Bootstrap procedure. Positive values indicate that social interactions/ToM have larger effect sizes. Abbreviations: Soc. – Social interactions. The table shows that social interactions and ToM have larger absolute effect sizes in the vast majority of comparisons, and most of them are statistically significant; no comparisons show significantly smaller absolute effect sizes of social interactions/ToM.

Table S2

Pearson’s Correlations between social cognitive features and ratings of social interactions and ToM across time

**Features**	**Social interactions**			**ToM**		
	**Audio**	**Text**	**All**	**Audio**	**Text**	**All**
Intentional actions	-.02	.11	.06	.03	.03	.03
All actions	-.10	.14	.06	.02	-.03	-.01
Biological motion	-.05	.09	.04	.11	-.07	-.003
Multi-person presence	.70	.81	.77	.26	.29	.28
Indoor – outdoor	.03	.20	.14	-.05	.27	.16
“Positive” label	.01	-.08	-.04	-.01	.01	-.001
“Neutral” label	.17	-.09	-.004	.01	-.19	-.12
“Negative” label	-.23	.12	.03	-.002	.19	.13
“Positive” probabilities	.03	-.07	-.05	.03	-.03	.004
“Neutral” prob.	.10	-.08	.01	-.05	-.13	-.08
“Negative” prob.	-.14	.11	.02	.03	.14	.08

Note. “Text” and “Audio” indicate correlations within Text and Audio narratives, respectively, and “All” indicates correlations across all narratives.

Character speaking and Physical interactions

Social interactions can be further divided into different types. Here we focused on two types, verbal interactions (character speaking) and non-verbal interactions (physical interactions). Following a previous study[23], we defined character speaking as where in the narratives there were actions or contents of one person talking or calling out to another person. We defined physical interactions as social interactions that involved bodily actions but no character speaking (i.e., character speaking and physical interactions should be exclusive). We manually annotated the two features by assigning a yes/no (1/0) label to each action part. For action parts that had both character speaking and physical interactions (e.g., “walk up to her partner to ask him”), we labeled them as character speaking but not physical interactions[23]. There were a few cases where the ratings for social interactions were high but the sentences were ambiguous in terms of character speaking or physical interactions, and we annotated them as having neither character speaking nor social interactions (e.g., “They formed a plan to infiltrate the base with Lucy’s brother”, “The two of them started spending a lot of time together and quickly developed a romantic relationship”). Across all narratives, about 25.9% of all time points were annotated as character speaking, and about 22.7% were annotated as physical interactions. The annotations of character speaking highly correlated with participants’ ratings of social interactions (rs = .50, .53 for Audio and Text modality, respectively; r = .52 across all narratives), while the annotations of physical interactions correlated very weakly (rs = .03, .06, .04). It suggests that the presence of character speaking, instead of physical interactions, might be the main feature that drove participants’ social interaction ratings. On the other hand, ratings of ToM did not correlate reliably with either character speaking (rs = .09, .14, .13) or physical interactions (rs = .15, .04, .08).

On the neural level, we first fit separate GLMs with regressor pairs (“character speaking” and “no character speaking”, or “physical” and “no physical”) that were boxcar curves covering the story periods with corresponding annotations and took the contrast within pairs as the neural correlates of processing character speaking or physical interactions. To investigate their commonalities and differences, we fit another combinatory model with both character speaking and physical interactions, as well as another regressor covering the story periods where there was no character speaking or physical interaction, which served as the contrast baseline for both character speaking and physical interactions. We found that whether to include both character

speaking and physical interactions in the same model had little effect on their neural correlates, as suggested by the large correlations between their effect maps with and without controlling for the other feature (all spatial *r*s > .91). In the combinatory model, their effect maps were moderately similar across modalities (spatial *rs* = .49, .51 for character speaking and physical interactions, respectively). Across modalities, character speaking activated bilateral PC, STS, dmPFC, IFG, and left TPJ (Figure S13a). Physical interactions activated bilateral TPJ, temporo-parieto-occipital junction, superior parietal lobule, premotor cortex, left supramarginal gyrus, and right dmPFC (Figure S13b). The shared activations were mainly in left TPJ, right IFG, and right dmPFC, and those regions might be the core social interaction processing regions. Character speaking activated larger regions on the left hemisphere than right, with physical interactions showing the opposite lateralization. Overall, the significant regions of the two types of social interactions overlapped only to a small extent (24.5% of the significant positive voxels activated by character speaking and 20.6% of those by physical interactions were shared with the other feature). It suggests that partially separable brain regions are involved in processing character speaking (verbal interactions) and physical interactions.

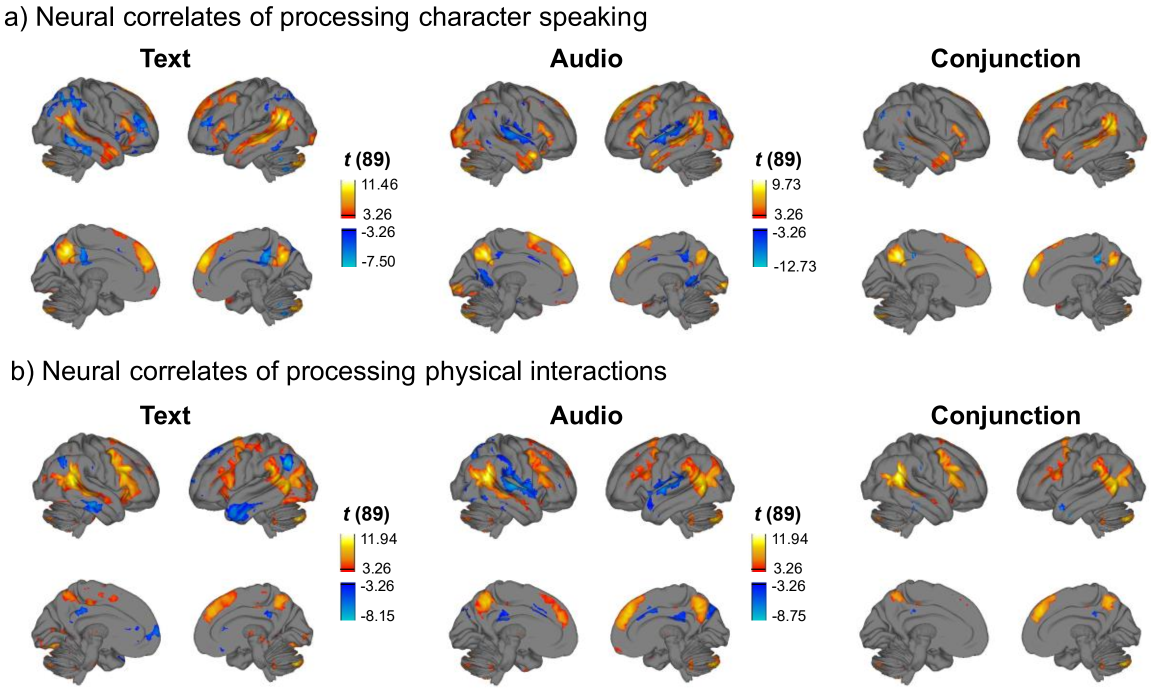

Figure S13. Neural correlates of processing character speaking (a) and physical interactions (b), thresholded at FDR $q < .01$. Their neural correlates partially overlapped and each included unique regions.

Linguistic features

Another group of features that may confound the effects of social interactions and ToM are the linguistic features that are embedded in the narrative texts. To test the possible confounding, we annotated several features by computational linguistic models and compared them with ratings of social interactions and ToM. We found that almost all correlations were very weak and not reliable (see Table S4 for a summary). It indicates that simple linguistic features cannot explain the variations in ratings of social interactions/ToM and cannot confound their effects. Below are details of each annotated feature.

Reading ease

We quantified the difficulty/ease of reading our narratives by two metrics: word frequency and number of syllables in each word. Word frequencies of all words in the narratives were found by the "wordfreq" python library[24], which returns a single frequency value for every English word based on various language sources. We found that word frequencies were not related to the medians of social interaction ratings (r = -.02) or ToM ratings (r = -.06) across all narratives used. Numbers of syllables of each word were counted by the "textstat" python library (https://pypi.org/project/textstat/). Results showed that numbers of syllables were not related to social interaction (r = .06) or ToM (r = .03) ratings. To rule out the possibility that ratings of social interactions and ToM had a "lag" behind the words that triggered them due to participants' response time, we incrementally shifted the time series of word frequencies and numbers of syllables backward and calculated the correlation for each shifted time series. We found that no shifted time series yielded larger correlations than the original ones.

Part-of-speech

The syntactic roles of words can be quantified by the part-of-speech (POS). We used the "flair" package in python[25] to predict the POS for each word in the narratives. The method implemented there is based on contextual word embeddings obtained from a character language recurrent neural network. On our narratives, the model yielded a high probability/confidence (>= 80%) for most predictions (3815 out of 3870 (98.6%)). In total, 30 unique types of POS tags were identified (for a full list, see Table S3). Most POS tags were relatively rare and could not support reliable estimations of their correlations with social interactions/ToM ratings. Thus, we focused on three individual tags that were found in at least 10% of all words ("NN", "IN", and "VBD"). We also made two larger tag groups: "all action verbs" were defined as the union of the tags of "VB", "VBD", "VBG", "VBN", "VBP", "VBZ", excluding the "be" verbs ("am", "is", "are", "was", "were", "be", "being", and "been"); "all nouns" were the union of "NN", "NNP", "NNS", and "NNPS". Across all narratives,

the above five tags correlated weakly with medians of social interaction ratings (all absolute rs < .01) or ToM ratings (all absolute rs < .04). The largest time-lag correlation is -.09 between ToM and NN when the time series of NN was shifted backwards 4.37 seconds, which was still trivial. It is worth noting that action verbs were not correlated with either social interaction ($r = .01$) or ToM ($r = .03$) ratings ($r = .07$ at maximum with time lags), which provides another piece of evidence that actions in the narratives were not a confounding factor for social interactions or ToM.

Then we tested whether a group of individual POS tags could jointly predict ratings of social interaction and ToM using 8-fold cross-validation. Specifically, in each fold, we held one narrative out as the test set and trained linear regression models that regressed social interaction or ToM ratings on POS tags in the remaining seven narratives. We restricted the POS tags used in training to those that were also present in the test narrative. Then we used the trained linear regression model to predict the social interaction or ToM ratings in the held-out narrative and calculated Pearson's correlations between predicted ratings and actual ratings. Results showed that across all narratives, the POS tags could not reliably predict social interaction ratings (average $r = .01$ (range [-.09, .07]) or ToM ratings (average $r = .06$, range [-.11, .17]). It indciates that POS cannot reliably explain the variance of social interaction or ToM ratings.

Table S3

All part-of-speech tags that were identified from the narratives

Tag	Meaning
CC	Coordinating conjunction
CD	Cardinal number
DT	Determiner
EX	Existential there
IN	Preposition or subordinating conjunction
JJ	Adjective
JJR	Adjective, comparative
JJS	Adjective, superlative
MD	Modal
NN	Noun, singular or mass
NNP	Proper noun, singular
NNPS	Proper noun, plural
NNS	Noun, plural
PDT	Predeterminer
PRP	Personal pronoun
PRP$	Possessive pronoun

RB	Adverb
RBR	Adverb, comparative
RBS	Adverb, superlative
RP	Particle
TO	to
VB	Verb, base form
VBD	Verb, past tense
VBG	Verb, gerund or present participle
VBN	Verb, past participle
VBP	Verb, non-3rd person singular present
VBZ	Verb, 3rd person singular present
WDT	Wh-determiner
WP	Wh-pronoun
WRB	Wh-adverb

Active/Passive voice

We manually annotated whether each action part in the narratives was written in the active voice or the passive voice. We found that a vast majority (96.1%) of actions in the narratives were in the active voice, so it is not feasible to get reliable estimates for its correlation with social interactions or ToM ratings, nor for its neural correlates.

Sentence complexity

Another feature related to reading ease is complexity of each sentence, quantified here by the Flesch-Kincaid Grade Level[26] calculated by the "textstat" library in python. The Flesch-Kincaid Grade Level is a weighted sum of the word frequencies, numbers of syllables, and sentence length, and puts larger weights on sentence length. Across all narratives, the score did not correlate meaningfully with medians of social interactions (r = .05) or ToM (r = .01) ratings. It indicates that sentence complexity cannot confound the effects of social interactions or ToM.

Semantic frames

Lastly, we considered possible semantic features conveyed by languages. We focused on the semantic "frames" defined by the FrameNet project[27]: A frame is a conceptual structure that describes a type of situation, object, or event along with its participants and props. Now there are more than 1,200 unique frames in the project. We used a state-of-the-art transformer-based model[28] to automatically identify semantic frames in our narratives. We found 275 unique frames in all narratives used, most of which appeared only once (42.5%) or twice (19.27%). To get reliable correlation estimates between any frames and social interactions or ToM ratings, we only considered the eight frames that appeared in at least 10% of all sentences: "Self_motion",

“Buildings”, “Perception_experience”, “Statement”, “Personal_relationship”, “People”, “Arriving”, and “Kinship”. Across all narratives, the frame “Statement” was moderately correlated with social interaction ratings (r = .32), while the absolute value of all the other correlations were below 0.2 and might not be meaningful.

The frame “Statement” is defined as containing verbs and nouns that communicate the act of a Speaker to address a Message to some Addressee using language. Clearly, this frame is one specific type of social interactions and overlaps a lot with character speaking (there is one exception in our narratives, though, where a character left a note to communicate some messages, which is using language without interactions). Thus, this frame should not be thought of as a confounder for the effects of social interactions.

Table S4

All linguistic features annotated, the methods of annotations, and their Pearson’s correlations with ratings of social interactions and ToM

**Linguistic features**	**Annotation method (package used)**	**Social interactions**			**ToM**		
		**Audio**	**Text**	**All**	**Audio**	**Text**	**All**
Reading ease – Word frequency	“wordfreq” in Python[24]	-.02	-.01	-.02	-.03	-.06	-.06
Reading ease – Number of syllables	“textstat” in Python (https://pypi.org/project/textstat/)	.03	.07	.06	.01	.04	.03
Part-of-speech (POS)	“flair” in Python[25]	<.05	<.04	< .01	<.03	<.06	< .04
		.01 (all POS prediction)			.06 (all POS prediction)		
Sentence complexity	“textstat” in Python	.09	.03	.05	-.03	-.0003	.01
Semantic frames – “Statement”	“frame-semantic-transformer” in Python[28]	.18	.39	.32	.16	.13	.14
Semantic frames – Other frames		<.12	< .09	< .09	<.11	<.13	< .12

Note. “Text” and “Audio” indicate correlations within Text and Audio narratives, respectively, and “All” indicates correlations across all narratives. Cells with a “<” indicate that the correlations were calculated between social interactions/ToM and more than one features (five POS tags and seven frames) and the absolute values of all correlations were below the value shown there.

Responses of ToM-selected regions to other social cognitive features

Quantifying social cognitive features other than social interactions and ToM made it possible to characterize how different brain regions responded to a series of different features and offer more insights of their functions. Here we focused on the "ToM-only" voxels (Figure 6) that did not respond to social interactions after controlling for ToM, because most of them were not found in earlier studies to be associated with ToM. We calculated a mean effect size (Cohen's *d*) for each feature in the ToM-only voxels and compared that against zero and with ToM. In both Text and Audio modality, ToM had significantly larger absolute effect size (Cohen's *d*s = .76, 1.25, respectively) in ToM-only voxels than all other features considered (all *p*s < .001) except character speaking in the Text modality (p = .09 under Bonferroni correction; Figure S14 and Table S5). Besides, among features other than social interactions and ToM, only multi-person presence, positive – negative, and physical interactions in the Audio modality had effect sizes significantly deviating from zero (all *p*s < .05 under Bonferroni correction); all other effect sizes were not different from zero (Figure S14 and Table S5). Those results supported that those voxels had selective responses to ToM but not other features.

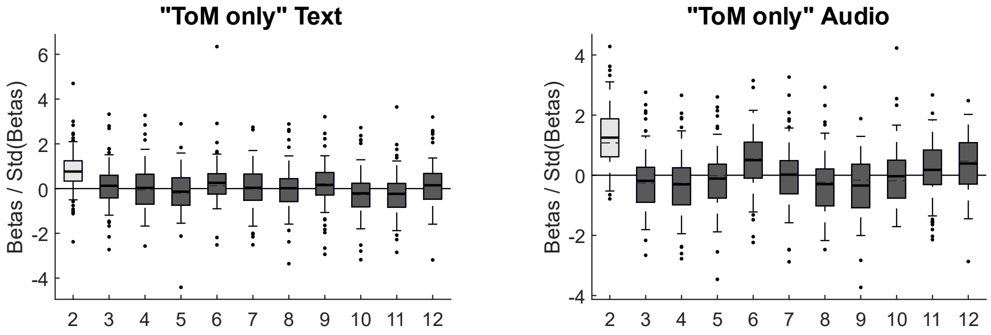

X-axis labels: 2 – ToM , 3 – Intentional actions, 4 – All actions, 5 – Biological motion
6 – Multi-person presence, 7 – Indoor vs. outdoor
8 – Positive vs. Negative, 9 – Positive vs. Neutral, 10 – Neutral vs. Negative
11 – Character speaking, 12 – Physical interactions

Figure S14. The effect sizes of ToM (light gray) and 10 other features in explaining neural activity in the "ToM-only" voxels (Figure 6). The *y* axes showed the individual mean regression coefficients (betas) divided by the standard deviation across participants (N = 90 participants for each box). The light gray box show effect sizes of ToM, and all other conventions follow Figure S12. The "ToM-only" voxels were not reliably activated by other features across modalities, and the effects of ToM were the strongest in both modalities.

Table S5

Effect sizes of ToM and other social cognitive features in ToM-only voxels

	ToM	Intentional actions	All actions	Biological motion	Multi-person presence	Indoor versus outdoor	Positive versus Negative	Positive versus Neutral	Neutral versus Negative	Character speaking	Physical interaction
Text	**0.76**	0.12	0.03	-0.14	0.26	0.03	0.01	0.16	-0.21	-0.24	0.15
Audio	**1.25**	-0.19	-0.30	-0.11	**0.50**	0.02	-0.30	**-0.34**	-0.03	0.18	**0.39**
ToM-Text	0	**0.64**	**0.73**	**0.62**	**0.50**	**0.73**	**0.75**	**0.60**	**0.55**	0.53	**0.61**
ToM-Audio	0	**1.07**	**0.95**	**1.14**	**0.75**	**1.23**	**0.96**	**0.91**	**1.22**	**1.07**	**0.86**

Note. Bold fonts indicate values that are significantly deviating from zero under the Bonferroni Corrected (22 comparisons) threshold of .05 (*p* values estimated from 10,000 Bootstrap samples). For most significant comparisons, an exact *p* value is not possible to estimate because of the precision limit of the Bootstrap procedure. In the first and second row, effect sizes of each feature in the Text and Audio modality were displayed. In the third and fourth row, the differences between the absolute effect sizes of ToM and those of each feature were in the Text and Audio modality were displayed; positive values indicate that ToM have larger absolute effect sizes.

fMRI effect tables

The tables below summarize the significant clusters/blobs of voxels in the main fMRI analyses reported in the main text. Without extra notes, all tables are organized by one cluster occupying three rows. The first row specifies the volume, peak coordinates (X, Y, and Z separately), and max *t* values. It also specifies which of the 16 cortical "Networks" from the resting-state cortical brain parcellation[29] has largest overlap (quantified by Dice coefficients) with the cluster; an entry of "Sub-cortex" means that the largest proportion of the cluster does not belong to any cortical networks. The third row contains names of the atlas regions which have >= 25% of the voxels with significant effects, with all names from the CANlab 2024 atlas (https://github.com/canlab/Neuroimaging_Pattern_Masks/tree/master/Atlases_and_parcellations/2024_CANLab_atlas). The second row contains "heuristic names" of those region names for easier interpretations, which are commonly used anatomy- or function-based brain region names that overlap with the atlas regions.

Table S6.

Regions associated with social interactions under both modalities

Cluster	Volume (mm^3)	X	Y	Z	Max t	Network
1	107200	-23	-77	-11	5.23	Sub-cortex

	auditory association cortex L, visual ventral L, parietal TPOJ L, parietal inferior lobule L, visual dorsal R, visual MT+ L, visual early L, visual dorsal L, visual ventral R, visual MT+ R, visual early R, Cblm cortex R, Cblm cortex L, Cblm vermis, temporal lateral L					
	Ctx STSvp L, Ctx V8 L, Ctx TPOJ2 L, Ctx PGi L, Ctx V3CD R, Ctx MST L, Ctx V4 L, Ctx MT L, Ctx V3CD L, Ctx V8 R, Ctx LO2 L, Ctx STSdp L, Ctx LO2 R, Ctx STV L, Ctx TPOJ1 L, Ctx V4 R, Ctx LO1 L, Ctx FST L, Ctx VMV3 L, Ctx LO1 R, Ctx V4t L, Cblm CrusII R, Cblm IX R, Cblm CrusII L, Ctx V3B L, Ctx PSL L, Cblm Vermis VIIIb, Cblm Vermis IX, Ctx PFm L, Cblm IX L, Ctx VVC L, Ctx STSva L, Ctx V3 R, Ctx PIT L, Ctx PHT L, Ctx V3 L, Ctx V1 L, Ctx FFC R, Cblm CrusI L					
2	137264	-11	30	40	5.16	Cortex_Default_ModeB
	cingulate dlPFC R, cingulate dlPFC L, cingulate ACC mPFC L, cingulate ACC mPFC R, somatomotor premotor L, CAU L, cingulate ventral frontal L, cingulate vlPFC L, insula anterior L, PUT L, temporal lateral L, somatomotor paracentral lobule L, somatomotor primary L					
	Ctx 8BL R, Ctx 8BL L, Ctx 9a L, Ctx 9m L, Ctx 9m R, Ctx 6d L, BG CAU VA L, Ctx SFL L, Ctx 8Av L, Ctx 9p L, Ctx 8C L, Ctx 6a L, Ctx 47s L, Ctx 9p R, Ctx 9a R, Ctx 8BM L, Ctx SFL R, Ctx 45 L, Ctx 44 L, Ctx 47l L, Ctx 8BM R, BG CAU body L, Ctx AVI L, BG PUT VA L, Ctx IFSp L, BG CAU DA L, Ctx d32 L, Ctx i6 8 L, BG PUT DP L, Ctx TGd L, Ctx s6 8 R, Ctx p9 46v L, Ctx 6ma L, Ctx s6 8 L, Ctx 3b L, Ctx AAIC L, Ctx 9 46d R, Ctx 55b L, Ctx 1 L, Ctx TGv L, Ctx IFJa L, Ctx TE1a L					
3	44768	52	20	8	7.03	Cortex_Fronto_ParietalA
	cingulate vlPFC R, cingulate dlPFC R, somatomotor premotor R, cingulate ventral frontal R, temporal lateral R, insula anterior R, auditory association cortex R					
	Ctx IFJa R, Ctx 8C R, Ctx 45 R, Ctx IFSp R, Ctx 44 R, Ctx 55b R, Ctx PEF R, Ctx 47s R, Ctx 47l R, Ctx 8Av R, Ctx TE1a R, Ctx TGd R, Ctx AVI R, Ctx FEF R, Ctx p9 46v R, Ctx STGa R, Ctx IFJp R, Ctx 6v R					
4	18176	56	-47	14	7.03	Cortex_Default_ModeC
	parietal TPOJ R, parietal inferior lobule R, auditory association cortex R					
	Ctx STV R, Ctx PGi R, Ctx STSvp R, Ctx TPOJ2 R, Ctx TPOJ3 R, Ctx STSva R, Ctx TPOJ1 R, Ctx STSdp R					
5	19488	6	-9	4	7.03	Sub-cortex
	Thal Posterior L, Thal Medial L, Midbrain R, CAU R, Midbrain L, Thal Ventral R, Thal Intralaminar L, PUT R, Thal Posterior R, Thal Intralaminar R, Thal Medial R, Thal Ventral L, Midbrain, VStriatum R, hypothalamus L, Thal Lateral L					
	Thal PuA L, Thal MD L, BStem mRt R, BG CAU body R, BStem RN R, BStem mRt L, Thal VL R, BStem STH L, Thal CL L, BG CAU VA R, BG PUT DA R, Thal PuA R, BG PUT DP R, Thal cIL R, BStem STH R, Thal MD R, Thal cIL L, Thal VL L, Thal VPL VPM L, Thal PuM L, Thal rIL R, Thal LGN L, BG CAU DA R, Thal MGN L, BStem CLi RLi, BStem VTA PBP L, Thal rIL L, BG BST SLEA R, Thal PuL L, BStem Shen Midb Rrd, hypothalamus posterior L, Thal LD L, BStem Shen Midb Lrd, Thal LP L, BStem SC R					
6	17880	-1	-59	40	3.76	Cortex_Default_ModeA
	cingulate posterior R, cingulate posterior L, parietal superior lobule L					
	Ctx 7m R, Ctx 31pd L, Ctx 7m L, Ctx 31pd R, Ctx 31pv L, Ctx PCV L, Ctx 7Pm L, Ctx d23ab L, Ctx POS2 R, Ctx d23ab R, Ctx POS2 L, Ctx v23ab R					
7	2200	-5	-13	54	4.73	Cortex_SomatomotorA
	somatomotor paracentral lobule L					
	Ctx 24dd L					

Note. This table is based on the results of the conjunction map of only social interaction regressors in the GLM. *t* values were calculated as the average of the *t* values in the effect maps under Text and Audio modality separately. Same applied for all tables below.

Table S7.

Regions associated with ToM engagement under both modalities

Cluster	Volume (mm^3)	X	Y	Z	Max t	Network
1	1376	-5	-59	-47	7.03	Sub-cortex
	Cblm cortex L					
	Cblm IX L					
2	506544	-3	-17	28	7.03	Multiple regions

	visual MT+ L, parietal superior lobule L, cingulate ACC mPFC L, cingulate dlPFC L, parietal inferior lobule L, visual dorsal L, cingulate dlPFC R, visual dorsal R, Thal Posterior L, cingulate posterior R, somatomotor paracentral lobule L, parietal superior lobule R, cingulate vlPFC R, somatomotor premotor L, Thal Intralaminar L, cingulate posterior L, somatomotor paracentral lobule R, parietal TPOJ R, visual MT+ R, cingulate ACC mPFC R, Midbrain R, visual ventral R, parietal TPOJ L, Thal Intralaminar R, cingulate vlPFC L, parietal inferior lobule R, Thal Posterior R, somatomotor premotor R, CAU L, auditory association cortex L, visual ventral L, Thal Medial L, somatomotor primary L, auditory association cortex R, cingulate ventral frontal L, Thal Ventral L, visual early L, insula anterior L, Thal Lateral L, Cblm cortex R, CAU R, hypothalamus L, PUT R, PUT L, hypothalamus R, Thal Ventral R, visual early R, Midbrain L, temporal lateral L, temporal lateral R, cingulate ventral frontal R, Cblm cortex L, Thal Medial R, Midbrain, somatomotor operculum L, VStriatum L, insula anterior R, Thal Anterior L, Thal Lateral R, GP L, VStriatum R					
	Ctx LO1 L, Ctx 7Pm L, Ctx 8BM L, Ctx 8BL L, Ctx 9p L, Ctx IP2 L, Ctx V3CD L, Ctx 8BL R, Ctx V3CD R, Thal PuA L, Ctx 7m R, Ctx 6ma L, Ctx i6 8 L, Ctx 7Pm R, Ctx MST L, Ctx s6 8 R, Ctx LIPd L, Ctx POS2 R, Ctx 9a L, Ctx 8Av R, Ctx 9p R, Ctx MT L, Ctx 45 R, Ctx 6a L, Thal cIL L, Ctx PGi L, Ctx POS2 L, Ctx 6ma R, Ctx 8Av L, Ctx STV R, Ctx MT R, Ctx 8C R, Ctx 8BM R, Ctx AIP L, Ctx 6d L, Ctx 31pd R, Ctx 7m L, Ctx SFL L, BStem STH R, Ctx i6 8 R, Ctx PCV L, Ctx V8 R, Ctx TPOJ2 L, Ctx 8C L, Thal rIL R, Ctx 44 L, Thal cIL R, Ctx 9m R, Ctx 9a R, Ctx LO3 R, Ctx 9m L, Ctx PFm L, Ctx PGi R, Thal PuA R, Ctx 6a R, Ctx 44 R, Ctx 46 L, BG CAU VA L, Ctx LO1 R, Ctx 31pd L, Ctx STSvp L, Ctx SFL R, Ctx V4t L, BStem mRt R, Ctx V8 L, Ctx p9 46v L, Ctx 24dd L, Ctx d32 L, Ctx MST R, Ctx FST R, Thal MD L, Ctx p9 46v R, BStem RN R, Ctx 6r L, Ctx PFt L, Ctx 7PC L, Ctx s6 8 L, Ctx PCV R, Ctx 2 L, Ctx 6d R, BG CAU DA L, Ctx TPOJ2 R, Ctx STSvp R, Ctx 47s L, Thal VPL VPM L, Ctx V4 L, Ctx SCEF L, Ctx FEF R, Ctx AVI L, Thal LD L, Ctx IP1 L, Ctx VMV3 L, Ctx LO2 R, Ctx STV L, Ctx V4t R, Ctx 6mp L, Cblm CrusII R, BG CAU body R, Ctx 7PL L, Ctx 6v R, Ctx PEF R, Ctx 45 L, Ctx 9 46d L, Ctx SCEF R, Ctx TPOJ3 R, Ctx d32 R, BG CAU DA R, Ctx FST L, Ctx 47l L, hypothalamus posterior L, Ctx 24dv L, BG PUT DP R, Thal CL L, Thal PuM L, BG PUT VA L, Ctx IFJa R, hypothalamus posterior R, Ctx p32pr L, Ctx 8Ad R, Thal VL R, Thal VL L, Ctx 23d L, Ctx 55b R, BG CAU body L, Ctx V4 R, BG CAU VA R, BStem STH L, Ctx TGd L, Ctx TE1a R, Ctx 31pv L, Ctx PF L, Ctx LIPv L, Thal PuL R, Ctx 47s R, Ctx TPOJ1 R, Cblm CrusII L, Thal rIL L, Ctx 1 L, Ctx 9 46d R, Ctx p47r L, Ctx PFm R, Thal VPL VPM R, Ctx DVT R, Ctx 47l R, BStem mRt L, Thal MD R, Ctx PGs R, BStem CLi RLi, Ctx 3b L, Thal PuL L, Thal LP L, Ctx 6v L, Ctx TGd R, Ctx LO3 L, hypothalamus anterior and tubular inferior R, Ctx 7PL R, Ctx OP1 L, Ctx V7 L, Ctx IFSp R, Ctx a9 46v L, BStem Shen Midb Rrd, Ctx 7Am L, Ctx DVT L, Ctx d23ab L, BG NAc L, Thal LGN R, Ctx PSL L, Cblm CrusI R, Ctx a32pr L, Ctx IP2 R, Ctx IPS1 R, Ctx VVC R, Ctx AVI R, Thal MGN L, Ctx PSL R, Ctx RSC L, Ctx 23c L, hypothalamus anterior and tubular inferior L, Ctx TGv L, Ctx STSva R, Ctx AIP R, Ctx 8Ad L, Thal MGN R, Ctx 46 R, Ctx d23ab R, BStem RN L, Ctx 31pv R, BG PUT DA R, Thal AV L, Ctx V3B L, BG PUT VP L, Thal LP R, Thal LD R, Ctx FFC R, BG GPe L, Ctx V3 R, Ctx a47r L, Ctx 4 L, Ctx VVC L, Ctx 7Am R, Ctx p10p L, Ctx v23ab R, Ctx V3B R, Ctx 5mv L, Ctx 3a L, Ctx RSC R, Ctx 24dd R, BG PUT DP L, Ctx 55b L, Ctx FEF L, Thal CL R, Ctx 24dv R, BStem VTA PBP R, Cblm CrusI L, BG BST SLEA R, Ctx 23d R, Ctx AAIC R					

Note. This table is based on the results of only ToM engagement regressors in the GLM.

Table S8.

Regions associated with ToM demands under both modalities

Cluster	Volume (mm^3)	X	Y	Z	Max t	Network
1	16680	-49	10	-23	3.95	Cortex_Default_ModeB
	cingulate vlPFC L, cingulate ventral frontal L, temporal lateral L					
	Ctx 45 L, Ctx 47l L, Ctx 44 L, Ctx TGv L, Ctx TGd L, Ctx 47s L					
2	6800	52	8	-35	3.95	Cortex_Limbic

	auditory association cortex R, temporal lateral R					
	Ctx STGa R, Ctx TE1a R, Ctx TGd R					
3	7240	32	-83	2	7.03	Cortex_Visual_Central
	visual ventral R, visual MT+ R, visual early R, visual dorsal R					
	Ctx VMV3 R, Ctx V8 R, Ctx LO1 R, Ctx V4 R, Ctx V3CD R					
4	7920	-27	-91	2	7.03	Cortex_Visual_Central
	visual ventral L, visual MT+ L, visual early L, visual dorsal L					
	Ctx VMV3 L, Ctx LO1 L, Ctx V4 L, Ctx V3CD L, Ctx VMV2 L					
5	6184	-53	-31	-5	7.03	Cortex_Default_ModeB
	auditory association cortex L					
	Ctx STSvp L, Ctx STSdp L					
6	4256	-49	-59	30	7.03	Cortex_Default_ModeB
	parietal inferior lobule L					
	Ctx PGi L					
7	14432	4	-65	38	5.98	Cortex_Fronto_ParietalC
	cingulate posterior R, cingulate posterior L					
	Ctx POS2 R, Ctx POS2 L, Ctx RSC L, Ctx RSC R					
8	20232	-11	36	50	3.48	Cortex_Default_ModeB
	cingulate dlPFC L, somatomotor paracentral lobule L, cingulate ACC mPFC L					
	Ctx SFL L, Ctx 9a L, Ctx 8BL L, Ctx 9p L, Ctx SCEF L, Ctx 9m L					
9	2128	-35	-57	50	6.92	Cortex_Fronto_ParietalA
	parietal superior lobule L, parietal inferior lobule L					
	Ctx LIPd L, Ctx IP2 L					

Note. This table is based on the results of only ToM demands regressors in the GLM.

Table S9.

Regions associated with social interactions controlling for ToM under both modalities

Cluster	Volume (mm^3)	X	Y	Z	Max t	Network
1	9320	24	-81	-37	4.44	Sub-cortex
	Cblm cortex R					
	Cblm CrusII R					
2	3744	4	-57	-43	3.72	Sub-cortex
	Cblm vermis, Cblm cortex R					
	Cblm Vermis IX, Cblm IX R					
3	15840	50	18	-21	7.03	Cortex_Limbic
	cingulate vlPFC R, temporal lateral R, auditory association cortex R, cingulate ventral frontal R, insula anterior R					
	Ctx 45 R, Ctx TGd R, Ctx TE1a R, Ctx STGa R, Ctx 47l R, Ctx 47s R, Ctx AVI R					
4	24456	-53	-35	4	7.03	Cortex_Default_ModeB
	auditory association cortex L, parietal inferior lobule L, parietal TPOJ L, temporal lateral L					

	Ctx STSvp L, Ctx PGi L, Ctx STSdp L, Ctx STSva L, Ctx TPOJ2 L, Ctx STV L, Ctx TE1a L, Ctx STSda L, Ctx TPOJ1 L					
5	7944	-23	-81	-35	7.03	Sub-cortex
	Cblm cortex L					
	Cblm CrusII L					
6	19672	-47	20	20	7.03	Cortex_Default_ModeB
	cingulate ventral frontal L, cingulate dlPFC L, cingulate vlPFC L, insula anterior L					
	Ctx 47s L, Ctx 8Av L, Ctx 8C L, Ctx 45 L, Ctx IFSp L, Ctx 44 L, Ctx 47l L, Ctx AVI L					
7	18744	-25	-91	-5	7.03	Cortex_Visual_Central
	visual ventral L, visual early L, visual MT+ L, visual dorsal L					
	Ctx V8 L, Ctx PIT L, Ctx V4 L, Ctx LO2 L, Ctx V3B L, Ctx V3CD L, Ctx V1 L					
8	10552	30	-89	-3	3.37	Cortex_Visual_Central
	visual early R, visual MT+ R, visual dorsal R, visual ventral R					
	Ctx V4 R, Ctx LO2 R, Ctx V3CD R, Ctx V3 R, Ctx V8 R					
9	2472	54	-27	-7	7.03	Cortex_Temporal_Parietal
	auditory association cortex R					
	Ctx STSvp R, Ctx STSva R, Ctx STSdp R					
10	1152	-3	-27	-1	4.18	Sub-cortex
	Midbrain L, Midbrain R					
	BStem mRt L, BStem SC R					
11	808	-7	-15	10	7.03	Sub-cortex
	Thal Medial L, Thal Posterior L					
	Thal MD L, Thal PuA L					
12	38064	-3	44	42	3.46	Cortex_Default_ModeB
	cingulate ACC mPFC L, cingulate dlPFC L, cingulate ACC mPFC R, cingulate dlPFC R					
	Ctx 9m L, Ctx 9a L, Ctx 8BL L, Ctx SFL L, Ctx 9m R, Ctx SFL R, Ctx 9p L, Ctx 8BL R, Ctx 8BM L, Ctx 9a R					
13	6688	-5	-57	36	7.03	Cortex_Default_ModeA
	cingulate posterior L, cingulate posterior R					
	Ctx 31pd L, Ctx 7m L, Ctx 31pv L, Ctx 7m R, Ctx d23ab L					

Note. This table is based on the results of both social interactions and ToM engagement regressors in the GLM.

Table S10.

Regions associated with ToM controlling for social interactions under both modalities

Cluster	Volume (mm^3)	X	Y	Z	Max t	Network
1	66848	34	-71	-5	6.65	Sub-cortex
	visual dorsal R, parietal TPOJ R, visual ventral R, visual MT+ R, parietal inferior lobule R, visual ventral L, visual early R, Cblm cortex R, Cblm cortex L, parietal superior lobule R					

	Ctx V3CD R, Ctx STV R, Ctx V8 R, Ctx MT R, Ctx LO3 R, Ctx LO1 R, Ctx PGi R, Ctx MST R, Ctx FST R, Ctx V4t R, Ctx V8 L, Ctx LO2 R, Ctx PFm R, Ctx V4 R, Cblm CrusII R, Ctx PGs R, Ctx VMV3 L, Ctx IP2 R, Ctx VVC R, Cblm CrusII L, Cblm CrusI R, Ctx AIP R, Ctx PGp R, Ctx TPOJ1 R					
2	7240	52	6	-35	5.95	Cortex_Limbic
	temporal lateral R					
	Ctx TE1a R, Ctx TGd R					
3	2392	-31	20	-23	7.03	Cortex_Default_ModeB
	cingulate ventral frontal L					
	Ctx 47s L					
4	9216	52	22	6	3.16	Cortex_Default_ModeB
	cingulate vlPFC R, cingulate ventral frontal R, somatomotor premotor R					
	Ctx 45 R, Ctx 44 R, Ctx 47l R, Ctx 6v R, Ctx 47s R					
5	13768	-7	-9	8	3.57	Sub-cortex
	Thal Posterior L, Thal Intralaminar R, Thal Intralaminar L, Thal Posterior R, CAU L, Midbrain R, Thal Medial L, Thal Ventral L, Midbrain, VStriatum L, Thal Ventral R, PUT L					
	Thal PuA L, Thal cIL R, Thal cIL L, Thal PuA R, BG CAU DA L, BStem mRt R, BG CAU VA L, Thal MD L, Thal VPL VPM L, Thal rIL R, BStem RN R, BStem CLi RLi, Thal PuM L, BG CAU body L, Thal PuL R, BG NAc L, Thal VPL VPM R, BG PUT VA L, Thal rIL L, Thal PuL L, BStem STH R					
6	4128	14	10	10	4.23	Sub-cortex
	CAU R					
	BG CAU DA R, BG CAU body R, BG CAU VA R					
7	1568	-53	-31	-5	6.90	Cortex_Default_ModeB
	auditory association cortex L					
	Ctx STSvp L					
8	279816	-11	-7	48	7.03	Multiple regions
	parietal superior lobule L, parietal superior lobule R, somatomotor paracentral lobule L, cingulate dlPFC L, cingulate dlPFC R, cingulate posterior R, cingulate ACC mPFC L, visual dorsal L, somatomotor premotor L, visual MT+ L, parietal inferior lobule L, somatomotor paracentral lobule R, cingulate posterior L, cingulate ACC mPFC R, somatomotor premotor R, cingulate vlPFC L, somatomotor primary L, visual early L, cingulate ventral frontal L, parietal TPOJ L					
	Ctx 7Pm L, Ctx 7Pm R, Ctx 6ma L, Ctx 9p L, Ctx 8BL R, Ctx 7m R, Ctx 8BM L, Ctx s6 8 R, Ctx 8BL L, Ctx 8Av R, Ctx i6 8 R, Ctx LIPd L, Ctx i6 8 L, Ctx POS2 R, Ctx V3CD L, Ctx 6a L, Ctx LO1 L, Ctx IP2 L, Ctx 6ma R, Ctx 31pd R, Ctx 8Av L, Ctx 6d L, Ctx PCV L, Ctx 8BM R, Ctx 7m L, Ctx SFL L, Ctx POS2 L, Ctx MST L, Ctx AIP L, Ctx 9a L, Ctx 6a R, Ctx 9p R, Ctx 46 L, Ctx PGi L, Ctx SFL R, Ctx MT L, Ctx 8C L, Ctx PFm L, Ctx p9 46v L, Ctx PCV R, Ctx 44 L, Ctx 24dd L, Ctx s6 8 L, Ctx 31pd L, Ctx 6r L, Ctx SCEF L, Ctx d32 L, Ctx 8Ad R, Ctx 2 L, Ctx 7PC L, Ctx p9 46v R, Ctx 6d R, Ctx IP1 L, Ctx PFt L, Ctx 6mp L, Ctx 7PL L, Ctx 9m R, Ctx FEF R, Ctx V4t L, Ctx 7PL R, Ctx 8C R, Ctx 9a R, Ctx 24dv L, Ctx 9m L, Ctx SCEF R, Ctx LIPv L, Ctx DVT R, Ctx FST L, Ctx 9 46d L, Ctx 1 L, Ctx d32 R, Ctx PF L, Ctx p32pr L, Ctx 45 L, Ctx 3b L, Ctx 7Am L, Ctx 6v L, Ctx 23d L, Ctx V4 L, Ctx 46 R, Ctx 7Am R, Ctx 9 46d R, Ctx p47r L, Ctx TPOJ2 L, Ctx 31pv R, Ctx 31pv L, Ctx 23c L, Ctx d23ab L, Ctx a32pr L, Ctx a9 46v L, Ctx 4 L, Ctx d23ab R, Ctx 3a L, Ctx DVT L, Ctx LO3 L, Ctx 5mv L, Ctx PSL L, Ctx 8Ad L, Ctx 24dd R					
9	1096	-49	-25	22	4.32	Cortex_SomatomotorB
	somatomotor operculum L					
	Ctx OP1 L					

Note. This table is based on the results of both social interactions and ToM engagement regressors in the GLM.

Texts of all narratives used in the study

In the texts below, each paragraph contains a narrative part that was constructed as one of the 36 dramatic situations[30] and presented in one experimental trial.

Narrative #1

One day at work, Margaret walked up to her partner, Al, to ask him to talk to the chief of staff at the local hospital where she worked as a nurse.

While waiting outside the chief of staff's office, Al heard nondescript shouting and yelling, there was a loud clatter in the room and then an eerie silence. An unfamiliar employee ran hastily out of the office with a terrified look on their face.

Al explained to the chief of staff that Margaret wanted to be excused from her absences last week. The chief of staff, Susan, revealed that she wanted to fire Margaret and never see her again. Susan and Al had been having an affair and she wanted Margaret "out of the picture". Al was conflicted but agreed it would be for the best.

Weeks passed and nobody had seen Margaret. Coworkers and friends searched the nearby city looking for her. One day on patrol in a nearby town, a police officer heard someone crying for help in the basement of a house and went in to investigate. Margaret yelled that she was being held captive and needed help.

In a rush, the police officer broke down the door and was attacked by a man in the house. Without hesitation, the officer shot the man and the man fell to the floor.

The officer rushed to the basement, untied Margaret, and took her to safety. After being notified of the situation, Al rushed to meet Margaret as to not raise any suspicion. When he arrived, he saw Margaret pointing and yelling at Susan who had been handcuffed and was talking to the police. Shortly after, the police took Susan away in a police car. Al was devastated to see her being taken away, as Margaret sneered with disdain.

Weeks later, Al went to county lockup to speak with Susan. After briefly catching up, he asked Susan about what happened at the house. Without realizing that their conversation was being recorded, Al foolishly mentioned their plans to get rid of Margaret. He even went as far to ask about what to do next. Within days, the two of them ended up confessing about their plot.

Shortly after, Margaret heard about Al's confession and infidelity on the local news. She was furious that she did not know what was going on and hurt that Al had been acting so selfishly behind her back.

Margaret went to a local bar to drown out her pain. After a few drinks at a quiet bar, she went to the back of the bar to use the facilities. On her way back, she heard someone approaching her from behind with a shuffled gait. She turned around and realized it was the man that was keeping her captive! Before she could make a sound, he covered her mouth with a rag and pulled her out the back of the bar – never to be seen again.

Narrative #2

Linda and Amy had been best friends since kindergarten, they even found homes on the same street in the forest on the edge of town. Their biggest fight had been over who gets the bigger slice of cake, until Amy married Dan. Linda

loathed Dan, she thought he was manipulative and had a violent temper. She only put up with him because Amy loved him.

This feeling of hate between Amy and Dan was mutual, Dan didn’t like how much time his wife spent with her and thought Amy was creating distance between their marriage. That’s why Dan decided to try and tarnish Linda’s reputation with his wife, in hopes it would get rid of her. He came home one afternoon and exclaimed that Linda had tried to kiss him. Amy was taken aback and immediately called Linda to confront her about these accusations. When Linda arrived, she denied having made a move on Dan, but discovered Dan had enlisted the help of his friend Will who said he “witnessed” it all happen.

Since it was their word against her own, she decided to go home and admit defeat. She knew how much it would hurt Amy to know the truth.

Months later Dan had taken up a mistress, he was growing bored with his marriage ever since he got rid of Linda and was only still with Amy for her family’s money. In a hotel room in a nearby town, Dan and his mistress, Nicole, conspired to secretly kill Amy while still obtaining her fortune.

They decided to have Dan invite her to the hotel room under the guise of a romantic date. Once she arrived, Nicole was there to tie her up. Once she was secured to a chair, Nicole demanded Amy's bank passwords. Amy obliged and gave her access to her bank accounts.

Nicole transferred all of Amy’s money into Dan’s bank account despite Amy’s pleading for her to stop.

Once successful, the mistress knocked Amy out with a forceful blow to the head. After seeing Amy’s body lying limp on the ground, she was suddenly filled with regret over her actions. Nicole quickly rushed her to the hospital, realizing she had only been jealous of Amy’s marriage with Dan and that she didn’t deserve to die.

Once Amy awoke from consciousness at the hospital, she was met by Linda. Linda rushed over as soon as she was called, finding out she was still Amy’s emergency contact. Nicole apologized and explained to Amy and Linda what had happened at the hotel. Amy was taken aback when she realized her husband's betrayal.

She decided it was best to let Dan run off with her money if that meant he was out of her life forever. Amy and Linda continued to be best friends, and years later she went on to marry a kind man with a loving family.

Narrative #3

It was just another Sunday afternoon for Lucy, she was on her way to the farmer’s market to pick up some produce. When she arrived at the old train depot that housed the market, she noticed two men fighting on the steps. Before she could call for help, one of the men crumpled to his knees. The other man dropped an object that appeared to be a knife and ran off. When Lucy approached the crime scene, she realized that the man who had been murdered was the Mayor and that he had a gunshot wound.

On the ground beside him was a pen that she had mistaken for a knife. Lucy looked for the gunman and saw a suspicious man in black with distinct pointy features darting into an alley across the street. Cops who were nearby chased the man who had fled and arrested him.

But Lucy knew the charged man was innocent, which meant the killer was free. Lucy vowed to find out who the real murderer was and to stop him at all costs.

The next day the mayor’s successor, Mr. Smith, a man who was very unpopular due to his strong ties to the oil companies, took office. After a long day of brainstorming about the mysterious killer, Lucy decided to attend his welcome ceremony in the city. During the mayor's speech, she noticed one of the security guards looked strangely

familiar. It dawned on her that this new mayor had motive to kill and that the man in black could've been the guard working for him.

Lucy decided to confront the mayor after his ceremony. But when she tried to accuse him, the security guards threatened to hurt her if she went to the police with her theory. Before she could say another word, a man pulled her from the crowd.

The man in question was another security guard of Mayor Smith's, who took Lucy into a hidden alleyway. His name was Max and he quickly explained that he was a part of the initial plot to kill the former Mayor, but he regretted his involvement.

He told Lucy about the Mayor's secret hideout in the forest that was used as a base for the Mayor's plots. They formed a plan to infiltrate the base with Lucy's brother Alex, a cop for the city, so they could find evidence of his crimes and arrest Mayor Smith. When the three arrived at the hideout, Max suggested they enter through the back. Once they snapped the lock off and opened the door, men grabbed hold of Lucy and Alex. The bodyguard in black came out and ordered the men to kill the cop immediately so he wouldn't talk. The man holding him instantly shot him in the chest.

Lucy pleaded with the guard to let them go so she could take her brother to the hospital, she said she would give up her mission against the mayor.

The guards laughed at her request and Lucy suddenly realized that they must have been tipped off on when they'd be at the hideout. She turned to Max and accused him of betraying them. Max shrugged and confessed to his disloyalty, he joined the other bodyguards in line and exclaimed neither of them were leaving this hideout alive. Resigned to her fate, Lucy closed her eyes and allowed the guards to kill her and any hope the former Mayor had of retribution.

Narrative #4

Luke always thought of himself as an honest man. That's why during his evening jog around the swamp, he pondered his recent affair with the receptionist at work. Luke looked up at the sky and asked God why he was so weak and unfaithful. He thought of the impact this would have on his family and told God of his conflicted feelings toward telling his wife Marta.

Suddenly, Luke heard a woman call out his name and when he turned around, he saw Carly, his receptionist, across the road with a gun in her hand. Carly ran to him and explained how she had followed him here to confess her love. She exclaimed if Luke didn't love her back that she would kill him and his family. Carly explained that the only way they could be together is to kill Marta.

Luke thought of his child and felt like this was his only option. He agreed to the plan and called his wife to meet him at the swamp to watch the sunset with him. When she arrived, Marta called out for her husband, but found Carly waiting for her. She immediately drew her gun and shot her in the chest, knocking her unconscious.

Luke ran to Marta and filled with regret over his decision to go along with Carly's plan, picked her up and rushed her to the hospital. The doctors tried to help Marta by taking her into emergency surgery but failed to save her life. Once the doctor called the time of death, Luke fell to his knees and sobbed over his rash decision.

He then saw Marta's brother Zach enter the hospital. Zach ran over to Luke and punched him. Zach screamed at Luke that it's his fault his sister is dead and that he knew about the affair. Luke quickly tried to explain that he felt horrible about Marta's death and that Carly had manipulated him into going along with her plan. Zach, still angry at his brother-in-law, realized his mistake in judgement and shifted his anger toward Carly.

After the tension with Zach cooled down, Luke thought of when he should call his son and inform him of his mother's

passing. He would be out of school any minute and should hear the news from him. Luke reflected on his decision and realized it was necessary to sacrifice his wife to ensure their child's safety, and that Marta had told him to above all else to protect him.

The police showed up at Carly's door a few days later, having matched the prints found on the murder weapon to hers. She was charged with first degree murder and life in prison. Years passed and she still thought of her love, Luke, and how she missed him. Until one day he showed up during visiting hours.

Luke explained he needed closure and told her how much he hated her for killing his wife. Carly was heartbroken over this because she had held on to this delusion that Luke still loved her after all these years. Once back in her cell she began to plot how to get back at Luke, she blamed him for her lifelong sentence and vowed to break out. She wanted to make him feel just as miserable as she did, and she knew the only way to do that was to take what he loved most, his son. She flirted with a guard who she knew was sweet on her and convinced him to help her escape.

Once outside the prison walls, she went to the school Luke's son attended and fooled the faculty into thinking she was his aunt. She then took the child and fled with him, never to be seen again.

Narrative #5

William and Johnny are brothers-in-law who had been cellmates for years. Both were facing the death penalty in the coming week. They had spent the last year planning a jailbreak together to avoid their looming death.

When the day came to make their escape, everything was going according to plan. They had successfully stolen the keys from the guard and had made their run toward the exit. As they approached the final gate, William looked over his shoulder and saw the guards catching up. He thought of his own freedom and being reunited with his wife and child. Just as the guards were about to capture them, William tripped Johnny and he fell to the floor. This distraction gave William just enough time to reach the exit and escape.

When William got to the street and met his wife, Shannon, with the car, she asked where her brother was. Seeing her husband's facial expression become grim, she quickly discovered William had left him behind.

Once the two returned home, Shannon decided to take a walk in the park next to their house to process the betrayal by her husband. She met up with her friend Robert, who had been helping her take care of her child and comfort her while William had been in prison. She shared her grievances with Robert as he hugged her and in a moment of weakness the two kissed.

When Shannon returned home, she discovered William had taken their child. He left a note explaining he had sacrificed Johnny, her brother, so he could provide their child with a father. He said that she would never understand so he had to leave.

Robert and Shannon quickly made a plan to get the child back from William.

They found out William had met up with his old criminal partner Aaron, who agreed to let William and their son stay at his beach house. Shannon remembered Aaron and knew William still in debt to Aaron financially; trying to repay Aaron is what initially landed William in jail. Shannon knew she had to get there quick before Aaron did anything to William, or their child.

Robert knew he needed to help Shannon and was willing to do what he knew Shannon couldn't, kill William, if it meant saving the child.

When they arrived at Aaron's beach house, William saw them immediately. He made a run for it with the child, and both Shannon and Robert quickly caught up to them. When the kid realized it was Robert and Shannon who had been

chasing them, he immediately ran away from William and into Robert's arms. William saw this and was heartbroken, wishing he had not missed so much of his child's life. He could see how happy his child was to see Robert. Aaron suddenly arrived and attempted to take the child from Robert. William lunged at him and yelled at Robert and Shannon to run away with the kid. William knew his child would be better off with them, so he stayed back to buy them time to escape.

Narrative #6

James's and Nancy's son Peter had been kidnapped recently. James told Nancy to stay at home and that he would search the whole town for the son, no matter how long it took. For days James had been searching all through town to find Peter, calling his name and hoping to hear his son's voice call back. One day while searching through an abandoned building, he finally heard his son yelling to him. He followed the voice and found his son tied to a chair.

After looking around to make sure no one else was there, he ran to his son, untied him and led him out of the building into the main part of town. While running through town on their way home, James and Peter ran into James's good friend Matthew. Matthew was so happy that James had found Peter, but he warned them that the kidnappers would soon see that Peter was missing and they would come for James and Peter this time. Matthew suggested that they hide out at his property in the city far from town.

James was furious that the kidnappers had taken his son and that they now had to flee and hide. He told Matthew to take Peter because he was going to find the kidnappers and take revenge on his own.

When James returned to their home, he saw his wife standing by the swamp near the house with two other men. Before he could react quick enough, he saw one of the men strike his wife and she fell to the ground.

James ran to Nancy's side and knelt before her crying while the other men were standing above him feeling no remorse. Suddenly, one of the men grabbed Nancy and despite the fact that James fought him, the man was able to overpower James and took Nancy and threw her body into the swamp.

James made his way to Matthew's house in the city to meet back up with his son. When he arrived, he told Matthew about Nancy's death. Matthew explained that he was sorry, but he thought that Peter should stay with Matthew and his wife because he needed a mother and father. In his state of sorrow and depression, he agreed that he did not have the capacity to take care of Peter and that it was best for him to stay with Matthew and his wife.

After the events of the last week or so, James felt he had suffered everything someone could.

Over the next couple weeks, the tragedies James had endured began to drive him mad and he spent the rest of his life alone and in agony and pain.

Narrative #7

Anna and her husband, Glenn, were in prison for robbing a convenience store. During their time in prison, Anna met Jeff, a man who was also convicted of robbery. The two of them developed a strong relationship, which turned into a romantic affair; Glenn was not aware of Anna's infidelity. One day Glenn saw Anna walking down a hallway by herself, so he followed her as she made her way to a storage closet. After she entered the closet, Glenn waited ten minutes and then went and knocked on the door. After a minute had gone by, Anna opened the door and was surprised to see Glenn standing before her. He pulled her out of the room and saw that her shirt was inside out and backwards. He grabbed her by the arms, started shaking her, and asked her what she was doing in the closet. His shaking became so violent that he shoved her to the ground. As he was about to hit her, Jeff came out of the closet and grabbed Glenn's hand before he could touch Anna again.

Jeff kneeled by Anna's side and took her hand. They discussed that something had to be done about Glenn.

Glenn could not believe what he had done and ran from them in a fit. Jeff and Anna avoided Glenn for the remainder of their sentences.

Six months later, all three of them were out of prison. Alone in the park, Glenn spoke to God and contemplated how he would atone for his sins and get his wife back.

Just then, he saw Anna walking by herself through the park; he had found her, and this was his opportunity to win her back.

Glenn ran to Anna and explained that he still loved her and had no intention to hurt her, but he wanted her back and would do anything to have her back in his life. Anna clutched her stomach and screamed that she was in pain. Glenn had no idea what was happening but swept her up and carried her to the hospital.

At the hospital, Glenn and Anna were met by Jeff, who had learned of her condition. The doctor explained that Anna was pregnant.

Anna and Jeff wanted to keep the baby but Glenn, who still wanted to be with Anna said that if the baby was Jeff's that she should terminate the pregnancy and be with him.

Anna turned to Glenn and told him that she didn't love him anymore, she loved Jeff, and that she and Jeff were going to keep the baby.

Narrative #8

Gloria and her brother, Jim, had been planning a month-long vacation together to a small town in Europe. The day before their trip, Gloria informed Jim that she couldn't go on the trip because her husband, Steve, no longer wanted her to go.

Jim understood but decided to go on the trip without her. When Jim did not return after a month, Gloria found out that Jim had been abducted.

Gloria was devastated and looked to Steve for support. Steve was not supportive in the way that she needed, so she turned to a coworker named Derek and he was there for her in every way possible. The two of them started spending a lot of time together and quickly developed a romantic relationship. Gloria and Derek decided that they wanted to be together and that Steve was in the way.

One day, Gloria and Derek were having dinner in the city together. Steve found them and confronted them about the affair. Steve pled for Gloria to stay with him, but she refused and demanded a divorce.

Steve turned to Derek and begged him not to take his wife from him; but Derek refused because he was in love with Gloria.

Gloria exclaimed that it was her decision and that she was going to choose Derek over Steve.

The next day, Gloria and Derek fled to Gloria's family's beach house to be alone. When they got to the beach house, they found Gloria's brother, Jim, tied up to a chair.

Apparently, he had never left for Europe but was being held captive. Gloria rushed to him, but as she was about to untie him, Jim's abductor seized Gloria and threw her to the ground.

In defense of Gloria and Jim, Derek grabbed a lamp and hit the abductor so hard that he died. Gloria released her brother and the two were finally reunited.

Divisions of sentence parts and action parts

Below we provide a full division of sentence parts used for annotating social interactions/ToM demands, and action parts used for rating intentional actions, accidental actions, and biological motion. Boundaries for action parts, by definition, are a subset of boundaries for sentence parts; in other words, one sentence part can have one or several action parts, but one action part cannot cross the boundary of sentence parts. The sentence parts that include more than one action part are primarily those that have one or two long noun clauses (they are objects in sentence parts, but action parts can have no object, so each noun clause can function as an independent action part), as well as the sentences that have participial clauses (they share and describe the subject in the main sentence part, so they are part of the sentence part but can be an independent action part). In the same texts as above, we put a “||” at each boundary of sentence parts and action parts, and a “|” at each boundary of only action parts.

Narrative #1

One day at work, Margaret walked up to her partner, Al, to ask him to talk to the chief of staff at the local hospital | where she worked as a nurse. ||

While waiting outside the chief of staff’s office, | Al heard nondescript shouting and yelling, || there was a loud clatter in the room and then an eerie silence. || An unfamiliar employee ran hastily out of the office with a terrified look on their face. ||

Al explained to the chief of staff | that Margaret wanted to be excused from her absences last week. || The chief of staff, Susan, revealed | that she wanted to fire Margaret and never see her again. || Susan and Al had been having an affair || and she wanted Margaret “out of the picture”. || Al was conflicted | but agreed it would be for the best. ||

Weeks passed || and nobody had seen Margaret. || Coworkers and friends searched the nearby city looking for her. ||One day on patrol in a nearby town, a police officer heard someone crying for help in the basement of a house | and went in to investigate. || Margaret yelled | that she was being held captive | and needed help. ||

In a rush, the police officer broke down the door || and was attacked by a man in the house. || Without hesitation, the officer shot the man || and the man fell to the floor. ||

The officer rushed to the basement, untied Margaret, and took her to safety. || After being notified of the situation, | Al rushed to meet Margaret as to not raise any suspicion. || When he arrived, | he saw Margaret pointing and yelling at Susan | who had been handcuffed and was talking to the police. || Shortly after, the police took Susan away in a police car. || Al was devastated to see her being taken away, || as Margaret sneered with disdain. ||

Weeks later, Al went to county lockup to speak with Susan. || After briefly catching up, | he asked Susan about what happened at the house. || Without realizing that | their conversation was being recorded, | Al foolishly mentioned their plans to get rid of Margaret. || He even went as far to ask about what to do next. || Within days, the two of them ended up confessing about their plot. ||

Shortly after, Margaret heard about Al’s confession and infidelity on the local news. || She was furious | that she did not know what was going on || and hurt | that Al had been acting so selfishly behind her back. ||

Margaret went to a local bar to drown out her pain. || After a few drinks at a quiet bar, she went to the back of the bar

to use the facilities. || On her way back, she heard someone approaching her from behind with a shuffled gait. || She turned around and realized | it was the man that was keeping her captive! || Before she could make a sound ||, he covered her mouth with a rag and pulled her out the back of the bar – never to be seen again.

Narrative #2

Linda and Amy had been best friends since kindergarten, || they even found homes on the same street in the forest on the edge of town. || Their biggest fight had been over who gets the bigger slice of cake, || until Amy married Dan. || Linda loathed Dan, || she thought | he was manipulative | and had a violent temper. || She only put up with him || because Amy loved him. ||

This feeling of hate between Amy and Dan was mutual, || Dan didn't like | how much time his wife spent with her || and thought | Amy was creating distance between their marriage. || That's why Dan decided to try and tarnish Linda's reputation with his wife, | in hopes it would get rid of her. || He came home one afternoon and exclaimed | that Linda had tried to kiss him. || Amy was taken aback || and immediately called Linda to confront her about these accusations. || When Linda arrived, | she denied having made a move on Dan, || but discovered | Dan had enlisted the help of his friend Will who said | he "witnessed" it all happen. ||

Since it was their word against her own, || she decided to go home and admit defeat. || She knew | how much it would hurt Amy to know the truth. ||

Months later Dan had taken up a mistress, || he was growing bored with his marriage || ever since he got rid of Linda || and was only still with Amy for her family's money. || In a hotel room in a nearby town, Dan and his mistress, Nicole, conspired to secretly kill Amy | while still obtaining her fortune. ||

They decided to have Dan invite her to the hotel room under the guise of a romantic date. || Once she arrived, || Nicole was there to tie her up. || Once she was secured to a chair, || Nicole demanded Amy's bank passwords. || Amy obliged and gave her access to her bank accounts. ||

Nicole transferred all of Amy's money into Dan's bank account | despite Amy's pleading for her to stop. ||

Once successful, the mistress knocked Amy out with a forceful blow to the head. || After seeing Amy's body lying limp on the ground, | she was suddenly filled with regret over her actions. || Nicole quickly rushed her to the hospital, || realizing she had only been jealous of Amy's marriage with Dan | and that she didn't deserve to die. ||

Once Amy awoke from consciousness at the hospital, || she was met by Linda. || Linda rushed over | as soon as she was called, || finding out she was still Amy's emergency contact. || Nicole apologized and explained to Amy and Linda what had happened at the hotel. || Amy was taken aback | when she realized her husband's betrayal. ||

She decided | it was best to let Dan run off with her money || if that meant he was out of her life forever. || Amy and Linda continued to be best friends, || and years later she went on to marry a kind man with a loving family.

Narrative #3

It was just another Sunday afternoon for Lucy, || she was on her way to the farmer's market to pick up some produce. || When she arrived at the old train depot that housed the market, || she noticed two men fighting on the steps. || Before she could call for help, || one of the men crumpled to his knees. || The other man dropped an object that appeared to be a knife and ran off. || When Lucy approached the crime scene, || she realized | that the man who had been murdered was the Mayor | and that he had a gunshot wound. ||

On the ground beside him was a pen that she had mistaken for a knife. || Lucy looked for the gunman | and saw a suspicious man in black with distinct pointy features darting into an alley across the street. || Cops who were nearby

chased the man who had fled and arrested him. ||

But Lucy knew | the charged man was innocent, || which meant the killer was free. || Lucy vowed to find out | who the real murderer was | and to stop him at all costs. ||

The next day the mayor’s successor, Mr. Smith, a man who was very unpopular due to his strong ties to the oil companies, took office. || After a long day of brainstorming about the mysterious killer, | Lucy decided to attend his welcome ceremony in the city. || During the mayor's speech, she noticed | one of the security guards looked strangely familiar. || It dawned on her | that this new mayor had motive to kill | and that the man in black could’ve been the guard working for him. ||

Lucy decided to confront the mayor after his ceremony. || But when she tried to accuse him, || the security guards threatened to hurt her || if she went to the police with her theory. || Before she could say another word, || a man pulled her from the crowd. ||

The man in question was another security guard of Mayor Smith’s, || who took Lucy into a hidden alleyway. || His name was Max || and he quickly explained | that he was a part of the initial plot to kill the former Mayor, | but he regretted his involvement. ||

He told Lucy about the Mayor's secret hideout in the forest that was used as a base for the Mayor’s plots. || They formed a plan to infiltrate the base with Lucy’s brother Alex, a cop for the city, || so they could find evidence of his crimes | and arrest Mayor Smith. || When the three arrived at the hideout, || Max suggested they enter through the back. || Once they snapped the lock off and opened the door, || men grabbed hold of Lucy and Alex. || The bodyguard in black came out and ordered the men to kill the cop immediately | so he wouldn’t talk. || The man holding him instantly shot him in the chest. ||

Lucy pleaded with the guard to let them go | so she could take her brother to the hospital, || she said | she would give up her mission against the mayor. ||

The guards laughed at her request || and Lucy suddenly realized | that they must have been tipped off on | when they’d be at the hideout. || She turned to Max and accused him of betraying them. || Max shrugged and confessed to his disloyalty, || he joined the other bodyguards in line and exclaimed | neither of them were leaving this hideout alive. || Resigned to her fate, Lucy closed her eyes | and allowed the guards to kill her and any hope the former Mayor had of retribution.

Narrative #4

Luke always thought of himself as an honest man. || That’s why during his evening jog around the swamp, he pondered his recent affair with the receptionist at work. || Luke looked up at the sky and asked God | why he was so weak and unfaithful. || He thought of the impact this would have on his family || and told God of his conflicted feelings toward telling his wife Marta. ||

Suddenly, Luke heard a woman call out his name || and when he turned around, | he saw Carly, his receptionist, across the road with a gun in her hand. || Carly ran to him and explained | how she had followed him here to confess her love. || She exclaimed | if Luke didn’t love her back | that she would kill him and his family. || Carly explained | that the only way they could be together is to kill Marta. ||

Luke thought of his child and felt like | this was his only option. || He agreed to the plan | and called his wife to meet him at the swamp to watch the sunset with him. || When she arrived, | Marta called out for her husband, || but found Carly waiting for her. || She immediately drew her gun and shot her in the chest, | knocking her unconscious. ||

Luke ran to Marta | and filled with regret over his decision to go along with Carly’s plan, | picked her up and rushed her to the hospital. || The doctors tried to help Marta by taking her into emergency surgery || but failed to save her life. || Once the doctor called the time of death, || Luke fell to his knees and sobbed over his rash decision. ||

He then saw Marta’s brother Zach enter the hospital. || Zach ran over to Luke and punched him. || Zach screamed at Luke | that it’s his fault his sister is dead | and that he knew about the affair. || Luke quickly tried to explain | that he felt horrible about Marta’s death | and that Carly had manipulated him into going along with her plan. || Zach, still angry at his brother-in-law, | realized his mistake in judgement || and shifted his anger toward Carly. ||

After the tension with Zach cooled down, || Luke thought of | when he should call his son and inform him of his mother's passing. || He would be out of school any minute || and should hear the news from him. || Luke reflected on his decision and realized | it was necessary to sacrifice his wife to ensure their child's safety, | and that Marta had told him to above all else to protect him. ||

The police showed up at Carly’s door a few days later, | having matched the prints found on the murder weapon to hers. || She was charged with first degree murder and life in prison. || Years passed || and she still thought of her love, Luke, | and how she missed him. ||Until one day he showed up during visiting hours. ||

Luke explained | he needed closure || and told her | how much he hated her for killing his wife. || Carly was heartbroken over this || because she had held on to this delusion | that Luke still loved her after all these years. ||

Once back in her cell she began to plot how to get back at Luke, || she blamed him for her lifelong sentence || and vowed to break out. || She wanted to make him feel just as miserable as she did, || and she knew | the only way to do that was to take what he loved most, his son. || She flirted with a guard who she knew was sweet on her || and convinced him to help her escape. ||

Once outside the prison walls, she went to the school Luke’s son attended || and fooled the faculty into thinking | she was his aunt. || She then took the child and fled with him, never to be seen again.

Narrative #5

William and Johnny are brothers-in-law who had been cellmates for years. || Both were facing the death penalty in the coming week. || They had spent the last year planning a jailbreak together to avoid their looming death. ||

When the day came to make their escape, || everything was going according to plan. || They had successfully stolen the keys from the guard || and had made their run toward the exit. || As they approached the final gate, || William looked over his shoulder and saw the guards catching up. || He thought of his own freedom and being reunited with his wife and child. || Just as the guards were about to capture them, || William tripped Johnny || and he fell to the floor. || This distraction gave William just enough time to reach the exit and escape. ||

When William got to the street and met his wife, Shannon, with the car, || she asked where her brother was. || Seeing her husband’s facial expression become grim, | she quickly discovered | William had left him behind. ||

Once the two returned home, || Shannon decided to take a walk in the park next to their house to process the betrayal by her husband. || She met up with her friend Robert, || who had been helping her take care of her child | and comfort her || while William had been in prison. || She shared her grievances with Robert || as he hugged her || and in a moment of weakness the two kissed. ||

When Shannon returned home, || she discovered | William had taken their child. || He left a note explaining | he had sacrificed Johnny, her brother, | so he could provide their child with a father. || He said | that she would never understand | so he had to leave. ||

Robert and Shannon quickly made a plan to get the child back from William. ||

They found out | William had met up with his old criminal partner Aaron, || who agreed to let William and their son stay at his beach house. || Shannon remembered Aaron || and knew William still in debt to Aaron financially; || trying to repay Aaron is what initially landed William in jail. || Shannon knew | she had to get there quick || before Aaron did anything to William, or their child. ||

Robert knew | he needed to help Shannon || and was willing to do | what he knew Shannon couldn't, kill William, || if it meant saving the child. ||

When they arrived at Aaron's beach house, || William saw them immediately. || He made a run for it with the child, || and both Shannon and Robert quickly caught up to them. || When the kid realized | it was Robert and Shannon who had been chasing them, || he immediately ran away from William and into Robert's arms. || William saw this | and was heartbroken, | wishing he had not missed so much of his child's life. || He could see | how happy his child was to see Robert. || Aaron suddenly arrived and attempted to take the child from Robert. || William lunged at him and yelled at Robert and Shannon to run away with the kid. || William knew | his child would be better off with them, || so he stayed back to buy them time to escape.

Narrative #6

James's and Nancy's son Peter had been kidnapped recently. || James told Nancy to stay at home | and that he would search the whole town for the son, | no matter how long it took. || For days James had been searching all through town to find Peter, | calling his name | and hoping to hear his son's voice call back. || One day while searching through an abandoned building, || he finally heard his son yelling to him. || He followed the voice | and found his son tied to a chair. ||

After looking around to make sure no one else was there, || he ran to his son, untied him and led him out of the building into the main part of town. || While running through town on their way home, || James and Peter ran into James's good friend Matthew. || Matthew was so happy | that James had found Peter, || but he warned them | that the kidnappers would soon see | that Peter was missing | and they would come for James and Peter this time. || Matthew suggested | that they hide out at his property in the city far from town. ||

James was furious | that the kidnappers had taken his son | and that they now had to flee and hide. || He told Matthew to take Peter || because he was going to find the kidnappers | and take revenge on his own. ||

When James returned to their home, || he saw his wife standing by the swamp near the house with two other men. ||

Before he could react quick enough, || he saw one of the men strike his wife || and she fell to the ground. ||

James ran to Nancy's side and knelt before her crying || while the other men were standing above him | feeling no remorse. ||

Suddenly, one of the men grabbed Nancy || and despite the fact that James fought him, || the man was able to overpower James || and took Nancy and threw her body into the swamp. ||

James made his way to Matthew's house in the city to meet back up with his son. || When he arrived, || he told Matthew about Nancy's death. || Matthew explained | that he was sorry, || but he thought | that Peter should stay with Matthew and his wife | because he needed a mother and father. || In his state of sorrow and depression, he agreed | that he did not have the capacity to take care of Peter | and that it was best for him to stay with Matthew and his wife. ||

After the events of the last week or so, James felt | he had suffered everything someone could. ||

Over the next couple weeks, the tragedies James had endured began to drive him mad || and he spent the rest of his life alone and in agony and pain. ||

Narrative #7

Anna and her husband, Glenn, were in prison for robbing a convenience store. || During their time in prison, Anna met Jeff, a man who was also convicted of robbery. || The two of them developed a strong relationship, || which turned into a romantic affair; || Glenn was not aware of Anna's infidelity. || One day Glenn saw Anna walking down a hallway by herself, || so he followed her || as she made her way to a storage closet. || After she entered the closet, || Glenn waited ten minutes || and then went and knocked on the door. || After a minute had gone by, || Anna opened the door || and was surprised to see Glenn standing before her. || He pulled her out of the room || and saw that her shirt was inside out and backwards. || He grabbed her by the arms, started shaking her, and asked her | what she was doing in the closet. || His shaking became so violent | that he shoved her to the ground. || As he was about to hit her, || Jeff came out of the closet and grabbed Glenn's hand || before he could touch Anna again. ||

Jeff kneeled by Anna's side and took her hand. || They discussed | that something had to be done about Glenn. ||

Glenn could not believe what he had done || and ran from them in a fit. || Jeff and Anna avoided Glenn for the remainder of their sentences. ||

Six months later, all three of them were out of prison. || Alone in the park, Glenn spoke to God and contemplated |how he would atone for his sins | and get his wife back. ||

Just then, he saw Anna walking by herself through the park; || he had found her, || and this was his opportunity to win her back. ||

Glenn ran to Anna and explained | that he still loved her | and had no intention to hurt her, || but he wanted her back || and would do anything to have her back in his life. || Anna clutched her stomach and screamed | that she was in pain. || Glenn had no idea what was happening || but swept her up and carried her to the hospital. ||

At the hospital, Glenn and Anna were met by Jeff, || who had learned of her condition. || The doctor explained | that Anna was pregnant. ||

Anna and Jeff wanted to keep the baby || but Glenn, who still wanted to be with Anna said | that if the baby was Jeff's | that she should terminate the pregnancy and be with him. ||

Anna turned to Glenn and told him | that she didn't love him anymore, || she loved Jeff, || and that she and Jeff were going to keep the baby. ||

Narrative #8

Gloria and her brother, Jim, had been planning a month-long vacation together to a small town in Europe. || The day before their trip, Gloria informed Jim | that she couldn't go on the trip | because her husband, Steve, no longer wanted her to go. ||

Jim understood || but decided to go on the trip without her. || When Jim did not return after a month, || Gloria found out | that Jim had been abducted. ||

Gloria was devastated | and looked to Steve for support. || Steve was not supportive in the way that she needed, || so she turned to a coworker named Derek || and he was there for her in every way possible. || The two of them started spending a lot of time together || and quickly developed a romantic relationship. || Gloria and Derek decided | that they wanted to be together | and that Steve was in the way. ||

One day, Gloria and Derek were having dinner in the city together. || Steve found them | and confronted them about

the affair. || Steve pled for Gloria to stay with him, || but she refused and demanded a divorce. ||

Steve turned to Derek and begged him not to take his wife from him; || but Derek refused || because he was in love with Gloria.

Gloria exclaimed | that it was her decision | and that she was going to choose Derek over Steve. ||

The next day, Gloria and Derek fled to Gloria's family's beach house to be alone. || When they got to the beach house, || they found Gloria's brother, Jim, tied up to a chair. ||

Apparently, he had never left for Europe || but was being held captive. || Gloria rushed to him, || but as she was about to untie him, || Jim's abductor seized Gloria and threw her to the ground. ||

In defense of Gloria and Jim, Derek grabbed a lamp | and hit the abductor so hard | that he died. || Gloria released her brother || and the two were finally reunited. ||

Supplementary References

1 Pelphrey, K. A., Morris, J. P. & McCarthy, G. Grasping the intentions of others: the perceived intentionality of an action influences activity in the superior temporal sulcus during social perception. *Journal of Cognitive Neuroscience* **16**, 1706–1716, doi:10.1162/0898929042947900 (2004).

2 Iacoboni, M. *et al.* Grasping the intentions of others with one's own mirror neuron system. *PLoS Biology* **3**, 79, doi:10.1371/journal.pbio.0030079 (2005).

3 Desmet, C. & Brass, M. Observing accidental and intentional unusual actions is associated with different subregions of the medial frontal cortex. *NeuroImage* **122**, 195–202, doi:10.1016/j.neuroimage.2015.08.018 (2015).

4 Kana, R. K. *et al.* Language and motor cortex response to comprehending accidental and intentional action sentences. *Neuropsychologia* **77**, 158–164, doi:10.1016/j.neuropsychologia.2015.08.020 (2015).

5 Deen, B. & McCarthy, G. Reading about the actions of others: biological motion imagery and action congruency influence brain activity. *Neuropsychologia* **48**, 1607–1615, doi:10.1016/j.neuropsychologia.2010.01.028 (2010).

6 Mele, A. R. & Cushman, F. Intentional action, folk judgments, and stories: Sorting things out. *Midwest Studies in Philosophy* **31**, 184–201, doi:10.1111/j.1475-4975.2007.00147.x (2007).

7 Isik, L., Koldewyn, K., Beeler, D. & Kanwisher, N. Perceiving social interactions in the posterior superior temporal sulcus. *Proceedings of the National Academy of Sciences of the United States of America* **114**, 9145– 9152, doi:10.1073/pnas.1714471114 (2017).

8 Landsiedel, J., Daughters, K., Downing, P. E. & Koldewyn, K. The role of motion in the neural representation of social interactions in the posterior temporal cortex. *NeuroImage* **262**, 119533, doi:10.1016/j.neuroimage.2022.119533 (2022).

9 Deen, B., Koldewyn, K., Kanwisher, N. & Saxe, R. Functional Organization of Social Perception and Cognition in the Superior Temporal Sulcus. *Cerebral Cortex* **25**, 4596–4609, doi:10.1093/cercor/bhv111 (2015).

10 Lahnakoski, J. M. *et al.* Naturalistic FMRI mapping reveals superior temporal sulcus as the hub for the distributed brain network for social perception. *Frontiers in Human Neuroscience* **6**, 233, doi:10.3389/fnhum.2012.00233 (2012).

11 Lee Masson, H. & Isik, L. Functional selectivity for social interaction perception in the human superior temporal sulcus during natural viewing. *NeuroImage* **245**, 118741, doi:10.1016/j.neuroimage.2021.118741 (2021).

12 Lee Masson, H., Chang, L. & Isik, L. Multidimensional neural represetations of social features during movie viewing. *Social Cognitive and Affective Neuroscience* **19**, 030, doi:10.1093/scan/nsae030 (2024).

13 Dufour, N. *et al.* Similar brain activation during false belief tasks in a large sample of adults with and without autism. *PloS One* **8**, 75468, doi:10.1371/journal.pone.0075468 (2013).

14 Brown, W. Some experimental results in the correlation of mental abilities1. *British Journal of Psychology* **3**, 296–322, doi:10.1111/J.2044-8295.1910.TB00207.X (1910).

15 Spearman, C. Correlation calculated from faulty data. *British Journal of Psychology* **3**, 271–295, doi:10.1111/j.2044-8295.1910.tb00206.x (1910).

16 Hasson, U., Nir, Y., Levy, I., Fuhrmann, G. & Malach, R. Intersubject synchronization of cortical activity during natural vision. *Science* **303**, 1634–1640, doi:10.1126/science.1089506 (2004).

17 Chen, J. *et al.* Shared memories reveal shared structure in neural activity across individuals. *Nature Neuroscience* **20**, 115–125, doi:10.1038/nn.4450 (2017).

18 McMahon, E., Bonner, M. F. & Isik, L. Hierarchical organization of social action features along the lateral visual pathway. *Current Biology: CB* **33**, 5035–5047 5038, doi:10.1016/j.cub.2023.10.015 (2023).

19 Epstein, R. & Kanwisher, N. A cortical representation of the local visual environment. *Nature* **392**, 598–601, doi:10.1038/33402 (1998).

20 Mormann, F. *et al.* Scene-selective coding by single neurons in the human parahippocampal cortex. *Proc Natl Acad Sci U S A* **114**, 1153–1158, doi:10.1073/pnas.1608159113 (2017).

21 Reddan, M. *et al.* Neural signatures of emotional intent and inference align during social consensus. *Nature Communications* **16**, 6156, doi:10.1038/s41467-025-59931-8 (2025).

22 Pérez, J. M. *et al.* pysentimiento: A Python Toolkit for Opinion Mining and Social NLP tasks. arXiv:2106.09462 (2021). <https://ui.adsabs.harvard.edu/abs/2021arXiv210609462P>.

23 Wolf, D. *et al.* Interpretation of Social Interactions: Functional Imaging of Cognitive-Semiotic Categories During Naturalistic Viewing. *Frontiers in Human Neuroscience* **12**, 296, doi:10.3389/fnhum.2018.00296 (2018).

24 Speer, R. *rspeer/wordfreq: v3.0 (v3.0.2*. (Zenodo, 2022).

25 Akbik, A., Blythe, D. & Vollgraf, R. in *Proceedings of the 27th International Conference on Computational Linguistics* 1638–1649 (2018).

26 Kincaid, J. P., Fishburne, R. P., Rogers, R. L. & Chissom, B. S. *Derivation of new readability formulas (automated readability index, fog count, and flesch reading ease formula) for Navy enlisted personnel (Report No. RBR-8-75*. (Chief of Naval Technical Training, Research Branch, 1975).

27 Baker, C. F., Fillmore, C. J. & Lowe, J. B. in *Proceedings of the 17th International Conference on Computational Linguistics -. the 17th international conference* (1998).

28 Chanin, D. *Open-source frame semantic parsing*. (In arXiv [cs.CL]. arXiv, 2023).

29 Yeo, B. T. T. *et al.* The organization of the human cerebral cortex estimated by intrinsic functional connectivity. *Journal of Neurophysiology* **106**, 1125–1165, doi:10.1152/jn.00338.2011 (2011).

30 Polti, G. *The thirty-six dramatic situations*. (Editor Company, 1917).
