# Supplementary material for: Common and distinct neural correlates of social interaction processing and theory of mind in narratives
Source: Nat Commun. 2026 Apr 4;17:4830. doi: 10.1038/s41467-026-71151-2 (PMC13223265; doi:10.1038/s41467-026-71151-2)
Supplement: Supplementary file 2 — Reporting Summary [file 41467_2026_71151_MOESM2_ESM.pdf]

nature portfolio

Corresponding author(s): Tor D. Wager

Last updated by author(s): Jan 22, 2026

Reporting Summary

Nature Portfolio wishes to improve the reproducibility of the work that we publish. This form provides structure for consistency and transparency in reporting. For further information on Nature Portfolio policies, see our Editorial Policies and the Editorial Policy Checklist.

Statistics

For all statistical analyses, confirm that the following items are present in the figure legend, table legend, main text, or Methods section.

n/a	Confirmed
☐	☒ The exact sample size (n) for each experimental group/condition, given as a discrete number and unit of measurement
☐	☒ A statement on whether measurements were taken from distinct samples or whether the same sample was measured repeatedly
☐	☒ The statistical test(s) used AND whether they are one- or two-sided<br>*Only common tests should be described solely by name; describe more complex techniques in the Methods section.*
☐	☒ A description of all covariates tested
☐	☒ A description of any assumptions or corrections, such as tests of normality and adjustment for multiple comparisons
☐	☒ A full description of the statistical parameters including central tendency (e.g. means) or other basic estimates (e.g. regression coefficient) AND variation (e.g. standard deviation) or associated estimates of uncertainty (e.g. confidence intervals)
☐	☒ For null hypothesis testing, the test statistic (e.g. F, t, r) with confidence intervals, effect sizes, degrees of freedom and P value noted<br>*Give P values as exact values whenever suitable.*
☐	☒ For Bayesian analysis, information on the choice of priors and Markov chain Monte Carlo settings
☐	☒ For hierarchical and complex designs, identification of the appropriate level for tests and full reporting of outcomes
☐	☒ Estimates of effect sizes (e.g. Cohen's d, Pearson's r), indicating how they were calculated

Our web collection on statistics for biologists contains articles on many of the points above.

Software and code

Policy information about availability of computer code

Data collection	In fMRI scan, Psychtoolbox (MATLAB, MathWorks) was used for stimulus presentation and response recording. In online experiments about social interactions, ToM, and multi-person presence, PsychoJS 2023.2.2 was used for stimulus presentation and response recording and Pavlovia was used for experiment hosting and data storage. In online experiments on intentional actions, all actions, and biological motion, Quatrics was used for stimulus presentation and data collection. In all online experiments, Prolific was used for participant recruiting. Stimuli and scripts to run experiments can be found at https://github.com/Zizhuang-Miao/Miao2025_social-tom/tree/main.
Data analysis	Open-source packages used include: CANlab neuroimaging analysis tools (https://github.com/canlab) in MATLAB R2022b, nltools 0.5.0 (https://nltools.org/) in Python 3.9.13, and fMRIPrep 21.0.2 (https://fmriprep.org/en/stable/). All scripts used to analyze the data can be found at https://doi.org/10.5281/zenodo.18315909.

For manuscripts utilizing custom algorithms or software that are central to the research but not yet described in published literature, software must be made available to editors and reviewers. We strongly encourage code deposition in a community repository (e.g. GitHub). See the Nature Portfolio guidelines for submitting code & software for further information.

Data

Policy information about availability of data

All manuscripts must include a data availability statement. This statement should provide the following information, where applicable:

- Accession codes, unique identifiers, or web links for publicly available datasets
- A description of any restrictions on data availability
- For clinical datasets or third party data, please ensure that the statement adheres to our policy

All data reported in the current study are openly accessible. All ratings and annotations of the narratives have been deposited in Zenodo [https://doi.org/10.5281/zenodo.18315909]91. The raw neuroimaging data (in the Nifti format and BIDS structure) can be found at [https://doi.org/10.18112/openneuro.ds005256.v1.1.0] and have been described extensively in Jung et al. (2025). The first-level beta images generated from raw neuroimaging data have been deposited in Zenodo [https://doi.org/10.5281/zenodo.18316150]. The Neurosynth dataset used in the present study is compiled on GitHub and can be found at https://github.com/canlab/Neuroimaging_Pattern_Masks/tree/master/Neurosynth_maps. The ToM group maps can be found at https://saxelab.mit.edu/use-our-theory-mind-group-maps/. The multimodal parcellation of the cerebral cortex50 can be found at https://github.com/canlab/Neuroimaging_Pattern_Masks/tree/master/Atlases_and_parcellations/2016_Glasser_Nature_HumanConnectomeParcellation.

Research involving human participants, their data, or biological material

Policy information about studies with human participants or human data. See also policy information about sex, gender (identity/presentation), and sexual orientation and race, ethnicity and racism.

Reporting on sex and gender	We confirm that we use the terms sex and gender in ways consistent with their definitions.<br>The study was designed to assess participants of male and female sex, as determined by self-report. It was not powered to systematically test effects of different genders to the degree that they diverge from self-reported sex. We did not consider sex or gender in the study design because we were studying human neural responses generalizable across sex/gender identity. We include self-reported sex in the metadata shared with the publication.
Reporting on race, ethnicity, or other socially relevant groupings	The participants in the neuroimaging study are primarily Caucasian-American, following the population demographics of the Upper Valley geographical area from which the sample was recruited. The participants in the online study are primarily self-reported as White, followed by Black, Asian, Mixed, and Others, which is the typical racial distribution of the online participant pool of Prolific. The study was not powered to investigate the differences among different racial or ethnic groups.
Population characteristics	In the neuroimaging study, data from 90 participants were included in analysis (55 females, 34 males, 1 other, 18-45 years old, M = 24.72, SD = 5.57). The inclusion criteria include: no current physical or mental disorders, English as native language or with comparable fluency, and normal or corrected-to-normal vision.<br>In the online study on social interactions and ToM, data from 231 participants were include in analysis (104 females, 127 males, 19-45 years old, M = 33.65, SD = 6.51). The inclusion criteria include: English as native language, no literacy difficulties, and an approval rate (proportion of valid data from studies they have taken part in) of over 95% on Prolific.<br>In the online study on multi-person presence, data from 103 participants were include in analysis (50 females, 53 males, 19-45 years old, M = 32.02, SD = 6.53). The inclusion criteria include: English as native language, no literacy difficulties, and an approval rate of over 98% on Prolific.<br>In the online study on intentional actions and all actions, data from 101 participants were include in analysis (52 females, 49 males, 19-45 years old, M = 34.38, SD = 6.62). In the online study on biological motion, data from 106 participants were include in analysis (51 females, 55 males, 19-45 years old, M = 33.27, SD = 6.64). All inclusion criteria were the same as that for multi-person presence.
Recruitment	In the neuroimaging study, participants were recruited from the area of the Upper Valley as well as the Dartmouth college student body, between December 2020 and July 2022. In the online studies, participants were recruited via Prolific (https://www.prolific.com/) between February 2024 and July 2025. All participants took part voluntarily, and small monetary incentive was provided.<br>This recruitment may incur the self-selection bias, because individuals with a strong interest in stories or brain-related studies may have been more likely to participate than the general population. Consequently, our sample may be more educated than the average population. While this may limit the external validity and generalizability of the findings to the broader population, it does not undermine the internal validity of the observed neural correlates of social interaction processing and theory of mind within this cohort.
Ethics oversight	All participants provided consent before participation. The institutional review board of<br>Dartmouth College approved to conduct the study and share the data for both the neuroimaging study (CPHS STUDY00031937) and the online studies (CPHS STUDY00032930).

Note that full information on the approval of the study protocol must also be provided in the manuscript.

Field-specific reporting

Please select the one below that is the best fit for your research. If you are not sure, read the appropriate sections before making your selection.

☐ Life sciences ☒ Behavioural & social sciences ☐ Ecological, evolutionary & environmental sciences

For a reference copy of the document with all sections, see nature.com/documents/nr-reporting-summary-flat.pdf

Behavioural & social sciences study design

All studies must disclose on these points even when the disclosure is negative.

Study description	The study is quantitative experimental.
Research sample	In the neuroimaging study, participants were recruited from the area of the Upper Valley as well as the Dartmouth college student body (55 females, 34 males, 1 other, 18-45 years old, M = 24.72, SD = 5.57). In all online studies, participants were recruited via Prolific and had to be US citizens or permanent residents (257 females, 284 males, 19-45 years old, M = 33.40, SD = 6.56). For the research questions of our interest concerning the neural correlates of basic social cognitive processes, our sample is representative of healthy adults because no evidence can support the effects of cultural and/or racial background.
Sampling strategy	We used convenience sampling method to recruit participants. We had sample sizes N = 90 for the neuroimaging study and total N = 541 for the behavioral studies. No statistical methods were used to pre-determine sample sizes. However, the sample size in the neuroimaging study is larger than the average for neuroimaging studies under similar topics (e.g., average n ≈ 22.6 per study in Arioli & Canessa (2019; https://doi.org/10.1002/hbm.24627) meta-analysis). The behavioral study produced highly reliable results (split-half correlations greater than 0.9 on continous measures) and we only used the group-level summary results for analysis, so the sample size is larger than sufficient.
Data collection	Neuroimaging data were collected in the fMRI scanner of Dartmouth Brain Imaging Center. Participants were alone in the scanning room during the task, but were introduced and screened for the experiment by two experimenters before the task. Behavioral data were collected online, where participants took part on their personal computers via the Internet. In the behavioral studies, the analysis focus was on the group-level summaries and relationships between different measures (social interactions, theory of mind, multi-person presence, and so on). In the neuroimaging study, all comparisons were within participant. Thus, there was no need for blinding in the study.
Timing	Neuroimaging data collection started in December 2020 and ended in July 2022. Behavioral data on social interactions and theory of mind were collected on February 5th-8th and May 13th-14th, 2024. Behavioral data on action-related features and multi-person presence was collected on July 1st to 8th, 2025.
Data exclusions	In the neuroimaging study, data from six participants were excluded from further analysis because of failures in fieldmap collections, which were essential for the susceptibility distortion correction step in fMRI preprocessing. In all behavior studies, data from 34 participants were excluded because they failed more than two attention check questions, and data from another five participants were excluded because they provided the same ratings throughout the experiment. All exclusion criteria were established prior to data collection.
Non-participation	The studies were one-shot and all participants who consented for participation completed the study. However, we lost the neuroimaging data from one participant and the behavioral data from three participants due to technical problems.
Randomization	In both neuroimaging and behavior studies on social interactions, theory of mind, and multi-person presence, the sequence of two narratives within one experimental run was counterbalanced across participants. In behavior studies on intentional actions, all actions, and biological motion, the orders of eight narratives were randomized for each participant. In behavioral studies, each participants was randomly allocated to one type of ratings.

Reporting for specific materials, systems and methods

We require information from authors about some types of materials, experimental systems and methods used in many studies. Here, indicate whether each material, system or method listed is relevant to your study. If you are not sure if a list item applies to your research, read the appropriate section before selecting a response.

Materials & experimental systems

n/a	Involved in the study	
☒	☐	Antibodies
☒	☐	Eukaryotic cell lines
☒	☐	Palaeontology and archaeology
☒	☐	Animals and other organisms
☒	☐	Clinical data
☒	☐	Dual use research of concern
☒	☐	Plants

Methods

n/a	Involved in the study	
☒	☐	ChIP-seq
☒	☐	Flow cytometry
☐	☒	MRI-based neuroimaging

Plants

Seed stocks

Report on the source of all seed stocks or other plant material used. If applicable, state the seed stock centre and catalogue number. If plant specimens were collected from the field, describe the collection location, date and sampling procedures.

Novel plant genotypes

Describe the methods by which all novel plant genotypes were produced. This includes those generated by transgenic approaches, gene editing, chemical/radiation-based mutagenesis and hybridization. For transgenic lines, describe the transformation method, the number of independent lines analyzed and the generation upon which experiments were performed. For gene-edited lines, describe the editor used, the endogenous sequence targeted for editing, the targeting guide RNA sequence (if applicable) and how the editor was applied.

Authentication

Describe any authentication procedures for each seed stock used or novel genotype generated. Describe any experiments used to assess the effect of a mutation and, where applicable, how potential secondary effects (e.g. second site T-DNA insertions, mosiacism, off-target gene editing) were examined.

Magnetic resonance imaging

Experimental design

Design type

Naturalistic viewing task

Design specifications

There was one session in the experiment with four runs. In each run, two narratives were played. Each narrative was divided into nine parts and each part was played in one trial. There was a jittered fixation period (a random integer from 2 to 8 seconds) between trials. The length of all trials ranged from 3 seconds to 52 seconds.

Behavioral performance measures

The behavioral data during fMRI scan were not analyzed or reported in the current study. However, the details are as follows: At the end of each trial, participants reported their current feeling and expectation about the story. They used the trackball to move the mouse cursor on a visual analog scale to the position corresponding to their rating, and then pressed the button to lock their rating. We checked the mouse trajectory and response time to make sure participants were performing the task as instructed.

Acquisition

Imaging type(s)

structural, functional

Field strength

3 Tesla

Sequence & imaging parameters

Structural images were acquired using high-resolution T1 spoiled gradient recall images (repetition time = 2000ms, echo time = 2.11ms, flip angle = 8°, field of view = 256mm, 320 × 320 matrix, phase encoding anterior » posterior) and were used for anatomical localization and warping to the standard Montreal Neurological Institute (MNI) space only.
Functional images were acquired with a multiband EPI sequence (repetition time = 460ms, echo time = 27.2ms, field of view = 220mm, multiband acceleration factor = 8, flip angle = 44°, 64 × 64 matrix, 2.7 × 2.7 × 2.7mm voxels, 56 interleaved ascending slices, phase encoding anterior » posterior).

Area of acquisition

A whole brain scan was used.

Diffusion MRI ☐ Used ☒ Not used

Preprocessing

Preprocessing software

Preprocessing was performed using fMRIPrep 21.0.2 (Esteban et al., 2019) based on Nipype 1.6.1 (Gorgolewski et al., 2011).
The parameters in calling the function included:
--ignore slicetiming
--dummy-scans 6
--bold2t1w-dof 9
--nprocs 12
--random-seed 2022
--skull-strip-fixed-seed
The preprocessed functional data were smoothed using a Gaussian kernel (Full Width at Half Maximum = 6mm) before further analysis.

Normalization

In preprocessing T1-weighted images, volume-based spatial normalization was performed through nonlinear registration with antsRegistration (ANTs 2.3.3), using brain-extracted versions of both T1w reference and the T1w template. In preprocessing functional images, the BOLD reference was co-registered to the T1w reference using bbregister (FreeSurfer) which implements boundary-based registration (Greve & Fischl, 2009). Co-registration was configured with nine degrees of freedom to account for distortions remaining in the BOLD reference.

Normalization template

ICBM 152 Nonlinear Asymmetrical template version 2009c (MNI152NLin2009cAsym, RRID:SCR_008796; TemplateFlow ID: MNI152NLin2009cAsym).

Noise and artifact removal

Head motion noise was controlled by adding six head motion parameters (x, y, and z translations, pitch, yaw, and roll), their derivatives, their squares and squares of derivatives into general linear models. Physiological noise was controlled by adding

mean CSF signals to the general linear models. Low-frequency noise (e.g., slow drifting) was controlled by high-pass filtering the data at 1/128 Hz.

Volume censoring

Volume censoring was performed after preprocessing in fMRIPrep on the run level and then single-volume level. We calculated the average signals at each voxel for each functional run and visualize them using nilearn 0.10.0; runs with significant data loss (e.g., very low signals in a large region) or aberrant data (e.g., data with the same magnitude across the whole brain) were manually marked and excluded from further analysis. In analyzing the data, we used nltools 0.5.0 to censor and exclude single volumes. Specifically, the program marked as outliers any volumes with a global signal or a framewise displacemen outside the +- 3 standard deviation range of those of all volumes in a run. Then we excluded the outlier volumes by entering each of them as an indicator function whose values were 1 at the time point of the outlier and 0 at all other time points in the general linear models.

Statistical modeling & inference

Model type and settings

We used the summary-statistic approach to build mixed-effect general linear models. Mass univariate model was applied in both first- and second-level analysis to calculate the voxel-level activations for social interactions and theory of mind. Bayes Factor analysis was performed on the second-level activation maps.

Effect(s) tested

The effects of interest include the regression coefficients between the normative ratings of social interactions and the neural activity at each voxel, and between the normative ratings of theory of mind and the neural activity at each voxel.

Specify type of analysis: ☐ Whole brain ☐ ROI-based ☒ Both

Anatomical location(s)

Three sets of anatomical locations were used: first from the association maps in neurosynth.org, second from the t maps or probabilistic maps shared in https://saxelab.mit.edu/use-our-theory-mind-group-maps/, and third from a probabilistic parcellation in Glass et al., (2016; https://github.com/canlab/Neuroimaging_Pattern_Masks/tree/master/Atlases_and_parcellations/2016_Glasser_Nature_HumanConnectomeParcellation)

Statistic type for inference

(See Eklund et al. 2016)

Voxel-wise

Correction

FDR correction

Models & analysis

n/a	Involved in the study
☒	☐ Functional and/or effective connectivity
☒	☐ Graph analysis
☐	☒ Multivariate modeling or predictive analysis

Multivariate modeling and predictive analysis

A set of support vector classification models were built as part of supplementary analysis. The inputs to the models were the vectorized whole-brain effect maps of social-interaction and no-social-interaction annotations, and the outputs were the labels of social interaction or no social interaction. Within-condition performance of the model was evaluated using five-fold cross validation, and cross-condition performance was evaluated by training the model using the entire dataset under one condition and testing it on another condition.
